# Eomes broadens the scope of CD8 T-cell memory by inhibiting apoptosis in cells of low affinity

**DOI:** 10.1371/journal.pbio.3000648

**Published:** 2020-03-17

**Authors:** Inga Kavazović, Hongya Han, Giulia Balzaretti, Erik Slinger, Niels A. W. Lemmermann, Anja ten Brinke, Doron Merkler, Jan Koster, Yenan T. Bryceson, Niek de Vries, Stipan Jonjić, Paul L. Klarenbeek, Bojan Polić, Eric Eldering, Felix M. Wensveen

**Affiliations:** 1 Department of Histology and Embryology, University of Rijeka, Rijeka, Croatia; 2 Center for Hematology and Regenerative Medicine, Department of Medicine, Karolinska Institutet, Karolinska University Hospital Huddinge, Stockholm, Sweden; 3 Brogelmann Research Laboratory, Department of Clinical Sciences, University of Bergen, Bergen, Norway; 4 Department of Clinical Immunology and Rheumatology, AMC Amsterdam, Amsterdam, the Netherlands; 5 Department of Experimental Immunology, AMC Amsterdam, Amsterdam, the Netherlands; 6 Institute for Virology and Research Center for Immunotherapy (FZI) at the University Medical Center of the Johannes Gutenberg University, Mainz, Germany; 7 Department of Immunopathology, Sanquin Research and Landsteiner Laboratory, AMC Amsterdam, Amsterdam, the Netherlands; 8 Department of Pathology and Immunology, University of Geneva, Geneva, Switzerland; 9 Department of Oncogenomics, AMC Amsterdam, Amsterdam, the Netherlands; National Jewish Medical and Research Center/Howard Hughes Medical Institute, UNITED STATES

## Abstract

The memory CD8 T-cell pool must select for clones that bind immunodominant epitopes with high affinity to efficiently counter reinfection. At the same time, it must retain a level of clonal diversity to allow recognition of pathogens with mutated epitopes. How the level of diversity within the memory pool is controlled is unclear, especially in the context of a selective drive for antigen affinity. We find that preservation of clones that bind the activating antigen with low affinity depends on expression of the transcription factor Eomes in the first days after antigen encounter. Eomes is induced at low activating signal strength and directly drives transcription of the prosurvival protein Bcl-2. At higher signal intensity, T-bet is induced which suppresses Bcl-2 and causes a relative survival advantage for cells of low affinity. Clones activated with high-affinity antigen form memory largely independent of Eomes and have a proliferative advantage over clones that bind the same antigen with low affinity. This causes high-affinity clones to prevail in the memory pool, despite their relative survival deficit. Genetic or therapeutic targeting of the Eomes/Bcl-2 axis reduces the clonal diversity of the memory pool, which diminishes its ability to respond to pathogens carrying mutations in immunodominant epitopes. Thus, we demonstrate on a molecular level how sufficient diversity of the memory pool is established in an environment of affinity-based selection.

## Abstract 2

This study shows that the diversity of the memory CD8 T-cell pool is regulated by the transcription factor Eomes, which drives transcription of the pro-survival protein Bcl-2. Genetic or therapeutic targeting of the Eomes/Bcl-2 axis reduces the clonal diversity of the memory pool.

## Introduction

The naïve CD8 T-cell pool consists of millions of clones, each unique based on its T-cell receptor (TCR). Upon pathogen encounter, a restricted number of antigen-specific cells are selected into the effector and memory pools. Their clonal diversity determines the scope of antigens that the population as a whole can recognize. There is an intrinsic difference in the way that the effector and memory pools must approach antigen, and this is reflected in their clonal selection strategies. Effector cells are faced with an actively replicating pathogen and benefit most from selection of only those clones with the highest specificity [1,2]. Typically, the effector pool against a given antigen is therefore dominated by only a few greatly expanded clones that bind the activating antigen with very high affinity [3,4]. In contrast, immunological memory must anticipate that pathogens are under immunological pressure to alter antigenic epitopes as they move through their host population [5]. The memory pool must therefore be more clonally diverse than the effector pool to sample a larger fraction of the potential pathogen-carried sequence space and thus have a higher chance of being able to recognize mutated pathogens. Increased clonal diversity is only possible if affinity-based selection is less stringent. Indeed, CD8 T-cell memory has been shown to be much more clonally diverse than the effector pool directed against the same antigen [4,6,7]. In line with this model, efficiency of the effector-cell pool is less when the affinity-based selection threshold of antigen-specific clones is reduced [2], whereas greater clonal diversity of the memory CD8 T-cell pool has been shown to be beneficial for the protection against rapidly mutating pathogens [4,8]. Selection of clones of high affinity into the effector pool depends on both increased proliferation [9] and an increased ability to access prosurvival cytokines [2]. How clones of both high and low affinity for the activating antigen are selected into the memory pool is currently unknown.

Experimental evidence indicates that the cumulative activating signal strength is a key determining factor for effector versus memory cell–fate decisions [10]. During priming, activated CD8 T cells integrate stimuli from the TCR, costimulatory molecules, and cytokines [11]. An increase or reduction of the cumulative signal intensity was shown to favor effector- or memory-cell formation, respectively [12,13]. Unknown is how this model integrates the concept of clonal diversity in its selection criteria. Statistical probability dictates that for any given antigen, the naïve pool will contain many more clones that bind it with low affinity than those that bind it with high affinity. Logic dictates that if low signal strength favors memory formation, any clone of high affinity would be outcompeted by multiple clones of low affinity into the memory pool. Yet clones that are most frequent during the effector phase also dominate memory directed against the same antigen [2,4,14]. At the same time, the memory pool does contain more clones than the effector pool directed against the same antigen, indicating that there are differences in selection criteria between these populations [3,4,6,7]. Previously, it was shown that effector-cell differentiation requires a certain activation threshold, which favors cells of high affinity by promoting proliferation and survival [2,9,15,16]. In contrast, even cells of very low affinity are capable of forming functional memory [9,17]. However, these studies were mostly performed using transfer of TCR transgenic cells with a single specificity, which largely negates the crucial aspect of clonal competition for memory-cell formation. Similar studies have shown that B cells expressing a transgenic receptor of low affinity for a given antigen are much less dominant in the presence of a competitor expressing an antigen receptor of high affinity [18,19]. How, under polyclonal conditions, CD8 T-cell clones of low affinity are allowed to make a significant contribution to the memory pool within an environment that favors outgrowth of clones of high affinity is unknown.

We find that memory precursors stimulated within a window of cumulative signal intensity maximally induce the transcription factor Eomes. Eomes directly drives expression of the prosurvival protein Bcl-2, providing a survival advantage for cells of submaximal affinity. Beyond this window, T-bet expression predominates, which inhibits Eomes-induced transcription of Bcl-2. The increased proliferative potential of clones activated with a TCR ligand of high affinity causes them to dominate the memory pool, whereas the survival advantage of cells of submaximal affinity ensures their persistence into memory, despite an environment of antigen affinity–based selection. These findings may be beneficial for the development of T cell–based vaccines with an increased scope.

## Results

### Submaximal TCR stimulation corresponds with higher expression of Eomes

**Fig 1:**
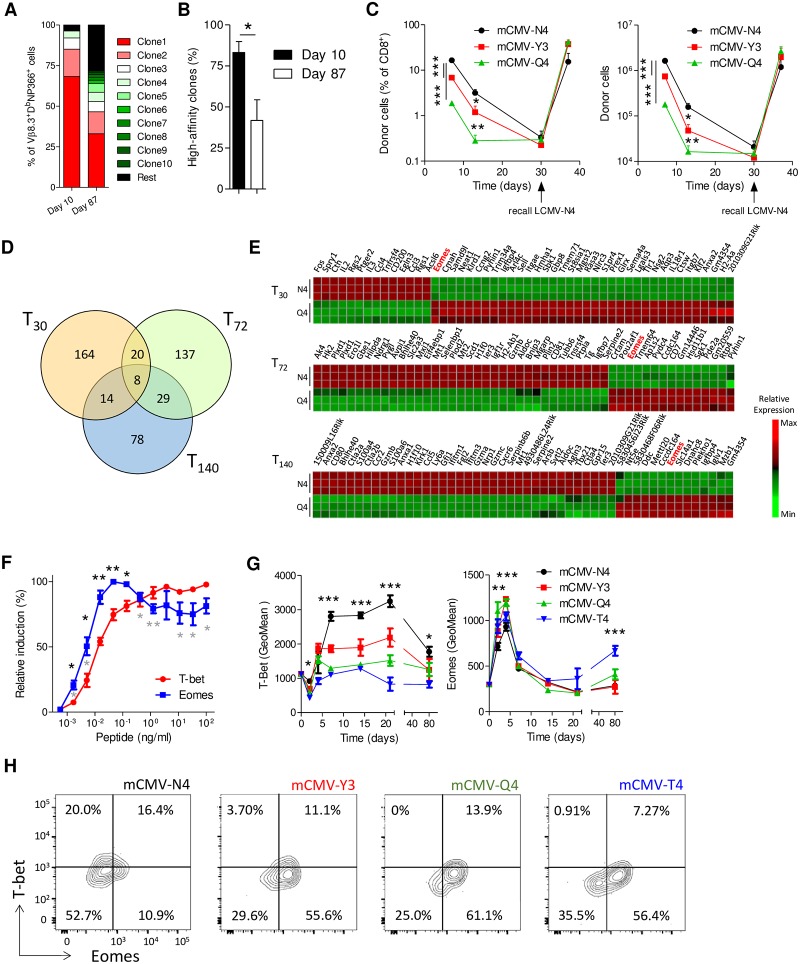
Submaximal TCR stimulation corresponds with higher expression of Eomes. (**A-B**) Mice were infected with influenza A strain PR/8/34. Ten (effector) and 87 (memory) days after infection, CD8^+^D^b^NP_366_^+^ cells were sorted, and their TCR was analyzed by NGS within the dominant Vβ8.3 family. (**A**) Frequency of clones as a percentage of total reads is given. Clones are ranked by relative size. Shown is the average clonal diversity from three mice per time point. (**B**) Cumulative contribution of the public high-affinity clones SGGS, SGGA, and SGGG (*n* = 3 per time point) to the total CD8^+^D^b^NP_366_^+^Vβ8.3^+^ repertoire. (**C**) CD45.1^+^ OT-1 cells (10^4^) were transferred to WT CD45.2^+^ recipients. After 24 hours, mice were infected with mCMV expressing the indicated peptides. Thirty days after infection, mice were reinfected with LCMV-N4. Shown is (left) the frequency and (right) absolute numbers of donor cells in spleen, determined by flow cytometry. (**D,E**) Purified OT-1 cells were primed for 30 hours with 1 ng/ml N4 or Q4 peptides and anti-CD28. Next, cells were washed and cultured for an additional 5 days with 50 ng/ml IL-15 to generate memory cells. RNA was isolated after 0, 30, 72, and 140 hours of culture (*n* = 3). (**D**) Venn diagram of differentially expressed genes between N4- and Q4-stimulated cells at indicated time points. (**E**) Heat map of top-50 differentially expressed genes at indicated time points. See also S1 Table. (**F**) Purified OT-1 cells were stimulated in vitro with anti-CD28 and indicated concentrations of N4 peptides. After 30 hours, relative induction of protein expression of T-bet and Eomes was determined by flow cytometry. Black asterisks indicate significant differences between T-bet and Eomes. Gray asterisks indicate significant differences of Eomes expression with cells stimulated at maximum signal (0.05 ng/ml). (**G,H**) CD45.1^+^ OT-1 cells were transferred in WT (CD45.2^+^) recipients. After 24 hours, mice were infected with mCMV expressing the indicated peptides. (**G**) Expression of T-bet and Eomes in donor cells in spleen was followed over time by flow cytometry. (**H**) Representative FACS plots show cells on day 2 after infection, with mCMV expressing the indicated peptides. Gated was for CD8^+^CD45.1^+^ cells. (**D,E**) Data from microarray analysis of three biologically independent samples, each pooled from 3–4 mice. Experiments show representative data from at least two (**A-C**) or four (**F-H**) independent experiments using 3–5 mice per group. In (**C,G**), ANOVA followed by Bonferroni posttesting was used; in (**B,F**), Student *t* test was used to analyze difference between groups. Shown are means ± s.e.m. *P < 0.05, **P < 0.01, ***P < 0.001. Values for each data point can be found in S1 Data. FACS, fluorescence-activated cell sorting; GeoMean, geometric mean; IL, interleukin; LCMV, lymphocytic choriomeningitis virus; mCMV, murine cytomegalovirus; N4, SIINFEKL; NGS, next-generation sequencing; Q4, SIIQFEKL; T4, SIITFEKL; TCR, T-cell receptor; WT, wild-type Y3, SIYNFEKL.

To investigate how the clonal diversity of effector and memory pools directed against the same antigen compare, mice were infected with influenza A strain PR/8/34 (PR8). Next, the clonal composition of CD8 T cells directed against the viral epitope NP366 was determined within the dominant [2] Vβ8.3^+^ family at the peak of the effector response (day 10) and at a memory time point (day 87). During the effector stage, the antigen-specific pool was dominated by three highly expanded clones (Fig 1A). The memory pool also contained a number of highly expanded clones, but these composed a much smaller fraction of the total population. Notably, on day 10 after infection, only 3.7% of the antigen-specific response consisted of clones with a frequency of <0.5%, whereas at memory time points, these clones composed 28.4% of the total pool. Infection of C57BL/6 (B6) mice with PR8 virus always results in the expansion of one to three clones with an identical (“public”) TCR sequence, and these have previously been shown to bind D^b^NP_366_ with high affinity [4,6,20]. As previously observed, at memory time points we found that the three public clones of high affinity (i.e., SGGS, SGGA, and SGGG) are significantly less dominant than they are in the effector pool (Fig 1B). Thus, we could confirm that the effector pool is more stringently selected for clones of high affinity than the memory pool directed against the same antigen.

To directly compare the impact of antigen affinity on the ability of CD8 T cells to contribute to the effector and memory pool, we transferred OT-1 TCR transgenic cells to naïve wild-type (WT) recipients and infected them with murine cytomegalovirus (mCMV) or *Listeria monocytogenes* (LM) strains expressing the high-affinity peptide SIINFEKL (N4) or altered peptide ligands (APLs), with the affinity hierarchy N4 > A2 (SAINFEKL) > Y3 (SIYNFEKL) > Q4 (SIIQFEKL) > T4 (SIITFEKL) [9]. In accordance with previous observations, we found that cells primed with high-affinity ligands formed many more effector cells on the peak of the T-cell response (day 7) (Fig 1C and S1A Fig). In contrast, whereas cells primed with high-affinity ligands formed more memory cells than did cells primed with low affinity, the difference was much smaller or even absent compared to the effector response after both mCMV and LM infection. Importantly, upon reinfection with lymphocytic choriomeningitis virus (LCMV)-N4, cells primed with high- and low-affinity ligands formed comparable recall responses (Fig 1C and S1A Fig), as reported previously [9]. These data indicate that cells activated with low-affinity ligands are prevented from making a large contribution to the effector pool, whereas their presence is mostly permitted in CD8 T-cell memory.

To investigate which factor controls the competitive fitness of antigen-specific clones activated with suboptimal affinity, we made use of an established in vitro model for memory formation [21]. OT-1 cells were stimulated with N4 peptides or APLs and low amounts of anti-CD28 for 30 hours, after which we cultured them for an additional 5 days with interleukin (IL)-15 only. On day 6 of culture, cells obtained phenotypic and functional features of memory cells and were able to mount robust recall responses 1 month after adoptive transfer in mice (S1B, S1C, S1D and S1E Fig). In this system, we stimulated cells with N4 or Q4 peptides, and gene expression profiles were generated at 0, 30, 72, and 140 hours. Comparison of differentially expressed genes at these three time points revealed eight genes to be differentially regulated consistently (Fig 1D and S1 Table). Of these, the transcription factor *Eomes* was more highly expressed at all three time points in Q4-stimulated cells, which was confirmed by quantitative polymerase chain reaction (qPCR) (Fig 1E and S1F Fig). In contrast, *Tbx21* (encoding for T-bet) was consistently lower expressed in Q4-stimulated cells. We therefore investigated how signal intensity relates to regulation of Eomes and T-bet in the context of avidity (titration range) and affinity (APLs) in vitro using the OT-1 model. We observed that Eomes is induced at lower activating signal strength than T-bet (Fig 1F). As T-bet levels reached a plateau, Eomes levels were mildly, though significantly, decreased. Notably, expression of CD122, a bona fide target of Eomes, highly correlated with expression of this transcription factor, whereas CD127 and CD25 did not (S1G Fig). Stimulation with APLs caused similar curves that were shifted to higher peptide concentrations (S1G and S1H Fig). Thus, early after CD8 T-cell activation, there is a window of stimulation strength in which Eomes-mediated transcription dominates over T-bet.

To confirm that clones primed with low-affinity ligands express more Eomes than cells primed with high-affinity ligands in vivo, OT-1 cells (CD45.1^+^) were transferred into WT recipients (CD45.2^+^), which were subsequently infected with mCMV expressing N4 or APLs. Indeed, T-bet induction positively correlated with affinity. In contrast, whereas all viral strains induced Eomes, its expression was significantly higher in OT-1 cells transferred to mice infected 2 and 4 days prior with mCMV expressing low-affinity APLs (Fig 1G and 1H, and S1I Fig). As previously reported [22], we observed a mild increase of Eomes expression levels at memory time points (Fig 1G and 1H). Thus, cells primed with low-affinity ligands induce higher levels of Eomes than cells primed with high-affinity ligands early after activation.

### Eomes promotes memory precursor formation early after activation

**Fig 2:**
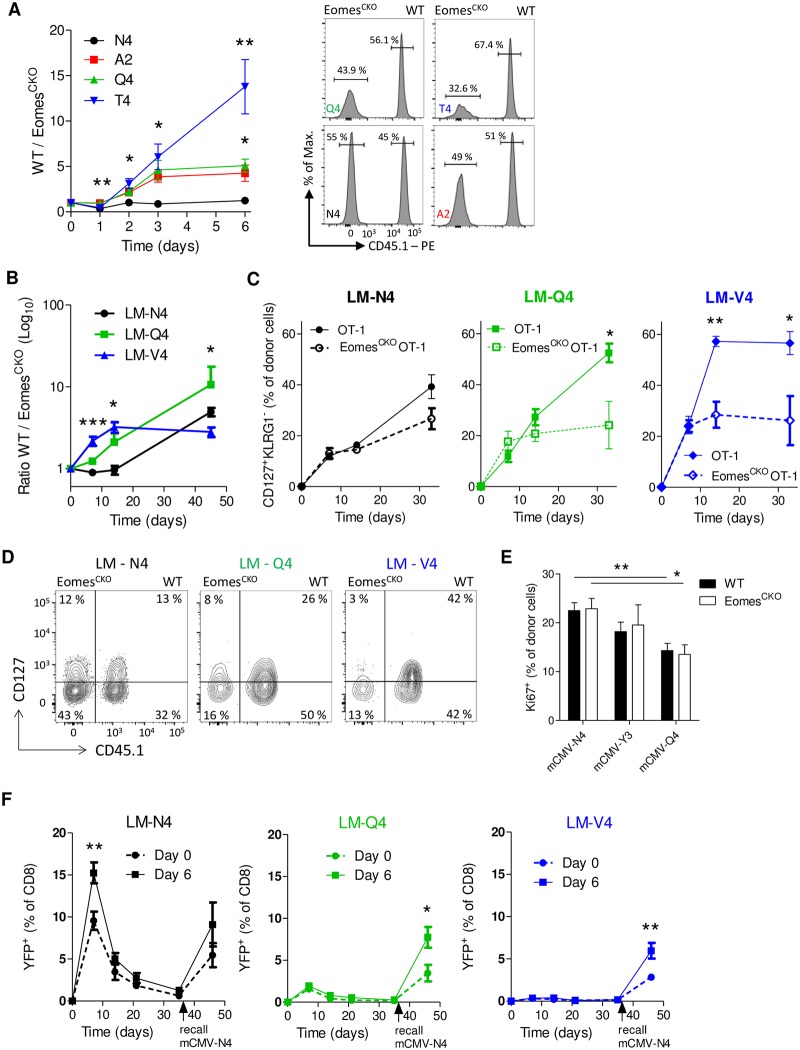
Eomes promotes memory precursor formation early after activation. (**A**) WT (CD45.1^+^) and Eomes^CKO^ (CD45.2^+^) OT-1 cells were mixed in a 1:1 ratio and stimulated with the indicated peptides. The ratio between cell populations was followed over time by flow cytometry. (**B-D**) OT-1 (CD45.1/2^+^) and Eomes^CKO^ OT-1 cells (CD45.2^+^) were mixed in a 1:1 ratio, and 10^4^ cells were injected into WT recipients (CD45.1^+^). Two days later, recipients were infected with LM-N4, LM-Q4, or LM-V4. (**B**) Ratio between WT and Eomes^CKO^ cells in blood over time. (**C**) Fraction of CD127^+^KLRG1^-^ cells among donor cells. (**D**) Representative FACS plots for day 14 were gated for CD8^+^CD45.2^+^ cells. (**E**) OT-1 (CD45.1/2^+^) and Eomes^CKO^ OT-1 cells (CD45.2^+^) were mixed in a 1:1 ratio, and 10^4^ cells were injected into WT recipients (CD45.1^+^). One day later, recipients were infected with mCMV-N4, mCMV-Y3, or mCMV-Q4. Seven days after infection, Ki67 expression was determined in splenic donor cells by flow cytometry. (**F**) Eomes^iCKO^ cells were transferred in WT recipients. Mice were infected with LM-N4, LM-Q4, or LM-V4 and injected with Poly(I:C) 0 or 6 days after infection. After 4 weeks, mice were reinfected with mCMV-N4. The fraction of eYFP^+^ donor cells was followed in the blood. Experiments show representative data from at least two (**B-F**) or four (**A**) independent experiments using 3–5 mice per group. In (**A,B,E**), ANOVA followed by Bonferroni posttesting was used; in (**C,F**), Student *t* test was used to analyze difference between groups. Shown are means ± s.e.m. *P < 0.05, **P < 0.01, ***P < 0.001. Values for each data point can be found in S1 Data. A2, SAINFEKL; Eomes^CKO^, Eomes^flox/flox^CD4^Cre^; Eomes^iCKO^, Eomes^FL/FL^OT-1^+/-^Rosa^Stop-EYFP^Mx1^Cre/+^; eYFP, enhanced yellow fluorescent protein; FACS, fluorescence-activated cell sorting; LM, *L*. *monocytogenes*; mCMV, murine cytomegalovirus; N4, SIINFEKL; Poly(I:C), polyinosine-polycytidylic acid; Q4, SIIQFEKL; T4, SIITFEKL; V4, SIIVFEKL; WT, wild-type; Y3, SIYNFEKL.

We hypothesized that Eomes is important for the persistence of CD8 T-cell clones of low affinity for the activating antigen into the memory pool. To investigate this, equal numbers of carboxyfluorescein succinimidyl ester (CFSE)-labeled WT and Eomes^flox/flox^ CD4Cre (Eomes^CKO^) OT-1 cells were mixed and stimulated in our in vitro model for memory formation. Low-affinity, but not high-affinity, ligands provided a selective advantage for WT cells compared to Eomes^CKO^ controls (Fig 2A). Analysis of CFSE dilution revealed no differences in the rate of proliferation between WT and Eomes-deficient cells (S2A Fig), both for cells stimulated with low- and high-affinity ligands and with ligands of low or high avidity, suggesting that Eomes is essential for survival of memory precursors of low affinity.

To confirm this in vivo, naïve WT OT-1 cells (CD45.1/2^+^) were mixed with an equal number of Eomes^CKO^ OT-1 cells (CD45.2^+^) and injected into WT recipients (CD45.1^+^). We infected recipients with LM expressing N4 or APLs, and cell ratios were followed in the blood. We observed that low-affinity ligands rapidly caused WT cells to dominate over Eomes-deficient cells, which was more pronounced after low- than high-affinity priming (Fig 2B). The importance of Eomes for long-term memory formation was strongest after LM-Q4 priming, which corresponds with an optimum curve of Eomes expression as observed after stimulation in vitro (Fig 1F). Importantly, we noticed that low-affinity, but not high-affinity, stimulation resulted in earlier memory formation, which was strongly impaired in cells lacking Eomes (Fig 2C and 2D). We then questioned whether Eomes deficiency affects proliferation of cells in vivo. Naïve WT OT-1 cells (CD45.1/2^+^) were mixed with an equal number of Eomes^CKO^ OT-1 cells (CD45.2^+^) and injected into WT recipients (CD45.1^+^). Next, recipients were infected with mCMV expressing N4 or APLs, and after 7 days, proliferation of donor cells was assessed in spleen. Whereas Ki67 negatively correlated with the affinity of the TCR ligand used for priming, no differences were observed between WT and Eomes^CKO^ cells (Fig 2E).

To investigate if and when Eomes impacts recall responses, we generated Eomes^flox/flox^Rosa^Stop-EYFP^Mx1^Cre^OT-1 (Eomes^iCKO^) mice in which elimination of Eomes could be induced by polyinosine-polycytidylic acid (Poly[I:C]) injection (Eomes^iCKO^) and followed by Cre-induced expression of an enhanced yellow fluorescent protein (eYFP) gene (S2B Fig). We transferred Eomes^iCKO^ OT-1 cells into WT recipients, which were subsequently infected with LM expressing N4 or APLs. Eomes deletion was induced either on the same day as primary infection with LM or on day 6 after adoptive transfer, after the peak of Eomes expression in vivo. After 6 weeks, recipient mice were reinfected with mCMV-N4, and the fraction of eYFP^+^ cells was determined in spleen. We found that early deletion of Eomes leads to a strong reduction in the ability to mount a recall response compared to late elimination of this gene after stimulation with low-affinity, but not high-affinity, ligands (Fig 2F).

Taken together, these data demonstrate that early after CD8 T-cell activation, Eomes promotes memory-cell formation, but its requirement inversely correlates with antigen affinity.

### Eomes promotes the clonal diversity of the memory CD8 T-cell pool

**Fig 3:**
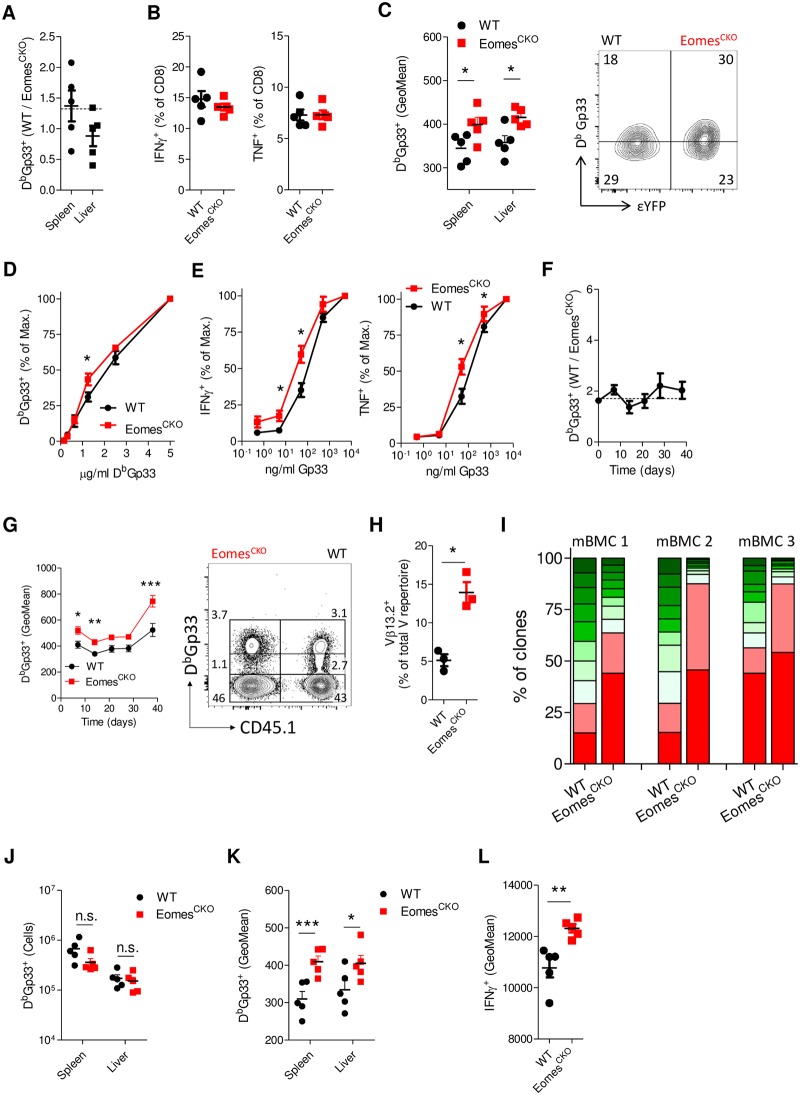
Eomes promotes the clonal diversity of the memory CD8 T-cell pool. mBMCs were infected with LCMV. (**A-E**) On day 7 after infection animals were analyzed. (**A**) Ratio between WT and Eomes^CKO^ D^b^Gp33^+^ T cells in indicated organs. Dashed line indicates ratio between total donor WT and Eomes^CKO^ CD8 T cells at day 0. (**B**) Frequency of IFNγ^+^ and TNF^+^ cells upon in vitro restimulation of splenocytes with Gp33 peptides. (**C**) Quantification of the GeoMean of tetramer binding of WT and Eomes^CKO^ D^b^Gp33^+^ CD8 T cells in spleen. Representative contour plot is gated for CD8^+^D^b^Gp33^+^ donor cells. Arbitrary gates indicate low and high tetramer binding. (**D**) Splenocytes were stained with increasing amounts of D^b^Gp33 tetramer. Percentage of tetramer^+^ cells relative to cells stained with 5 μg/ml is shown. (**E**) Splenocytes were stimulated in vitro with increasing amounts of Gp33 peptides. Percentage of IFNγ^+^ and TNF^+^ cells relative to cells stimulated with 5 μg/ml is shown. mBMCs were generated using WT (CD45.1^+^) and Eomes^CKO^ (CD45.2^+^EYFP^+^) cells in WT (CD45.2^+^) recipients. Reconstituted animals were infected with LCMV and at indicated time points cells were analyzed by flow cytometry. (**F,G**) Analysis of D^b^Gp33^+^ WT and Eomes^CKO^ CD8 T cells in blood at indicated time points after infection. (**F**) Ratio between donor D^b^Gp33^+^CD8^+^ WT and Eomes^CKO^ T cells over time. Dashed line indicates ratio between total donor WT and Eomes^CKO^ CD8 T cells at day 0. (**G**) Quantification of the GeoMean of D^b^Gp33 binding of WT and Eomes^CKO^ tetramer^+^ CD8 T cells. Representative FACS plot shows cells at day 38 after infection. Gated is for CD8^+^ donor cells. Arbitrary gates indicate no, low, and high tetramer-binding CD8^+^ T cells. (**H,I**) Thirty days after infection, WT and Eomes^CKO^ CD8^+^D^b^Gp33^+^ cells were sorted and analyzed by TCR sequencing. (**H**) Shown is the contribution of clones within the Vβ13.2^+^ family as a frequency of the total response. (**I**) Contribution of individual clones to the Vβ13.2 family. Each color represents one clone. (**J-L**) On day 41 after infection, mice were reinfected with LM-Gp33. After 5 days, antigen-specific responses were analyzed. (**J**) Number of D^b^Gp33^+^ WT and Eomes^CKO^ CD8 T cells. (**K**) Quantification of the GeoMean of D^b^Gp33 binding of tetramer^+^ CD8 T cells. (**L**) Splenocytes were stimulated in vitro with Gp33 peptides and cytokine production was determined by FACS. Student *t* test was used to analyze differences between groups. Shown are representative plots of at least two experiments using 3–6 mice per group. Shown are means ± s.e.m. *P < 0.05, ***P < 0.001. Values for each data point can be found in S1 Data. Eomes^CKO^, Eomes^flox/flox^ CD4Cre; FACS, fluorescence-activated cell sorting; GeoMean; geometric mean; Gp33, KAVYNFATC; IFNγ, interferon gamma; LCMV, lymphocytic choriomeningitis virus; LM, *L*. *monocytogenes*; mBMC, mixed bone marrow chimera; n.s., not significant; TCR, T-cell receptor; TNF, tumor necrosis factor; WT, wild-type; YFP, yellow fluorescent protein.

Whereas the OT-1 system is a powerful tool, it does not recapitulate the full complexity of interclonal competition after antigen encounter. To investigate the impact of Eomes deficiency on memory formation in a more physiological setting in vivo, we generated mixed bone marrow chimeras (mBMCs) containing Eomes^CKO^ (CD45.2^+^) and WT (CD45.1^+^) cells in an equal proportion. Animals were infected with LCMV, and the antigen-specific response was followed over time. We did not observe a difference in the magnitude of the effector response or in the ability of cells to produce cytokines and only found a small reduction in KLRG1 expression of Eomes^CKO^ cells (Fig 3A and 3B, and S3A Fig), as previously reported [23]. Notably, we observed a modest but significant increase in the overall antigen affinity of virus-specific Eomes^CKO^ cells, as demonstrated by higher tetramer binding and more cytokine production after restimulation with limiting peptide concentrations (Fig 3C, 3D and 3E). This effect was not specific for LCMV, as similar observations were made after infection of mBMCs with mCMV-N4 (S3B, S3C, S3D, S3E, S3F, S3G and S3H Fig). Key proteins involved in TCR signaling were not differentially expressed in Eomes^CKO^ cells either after LCMV or mCMV-N4 infection (S3I and S3J Fig).

In contrast to previous reports [24], Eomes deficiency did not result in a loss of memory cells after LCMV infection (Fig 3F). Differences in antigen affinity increased over time, as effector cells were progressively lost (Fig 3G, and S4A and S4B Fig). Similar observations were made after mCMV-N4 infection (S4C, S4D and S4E Fig). Notably, Eomes^CKO^ cells expressed higher levels of T-bet early after infection (S4E Fig) and higher levels of CD5, a marker previously associated [25] with high affinity (S4F Fig). These findings suggest that in absence of Eomes, memory clones of low affinity for their activating ligand fail to survive during memory formation. To confirm this hypothesis, mBMCs were infected with LCMV, and after 30 days, the clonal composition of WT and Eomes^CKO^ D^b^Gp33^+^ CD8^+^ T cells was determined within the dominant Vβ13.2^+^ family. Though some variance within Vβ usage was observed, we found that Eomes deficiency resulted in a further skewing of the response toward the Vβ13.2^+^ family (Fig 3H). Importantly, within the Vβ13.2^+^ family, we observed that the Eomes^CKO^ memory-cell pool was dominated by one or two highly expanded clones, whereas the WT memory pool within the same mouse was much more diverse (Fig 3I). Thus, Eomes deficiency results in a reduction of clonal diversity within the CD8 memory T-cell pool. Eomes-deficient memory cells were not functionally impaired, as they generated a recall response of similar magnitude after reinfection with LM expressing KAVYNFATC (Gp33), the immunodominant epitope of LCMV (Fig 3J). Differences in antigen affinity were retained after recall and even caused an increase in cytokine production after in vitro restimulation (Fig 3K and 3L and S4G and S4H Fig).

In summary, Eomes promotes the clonal diversity of the memory CD8 T-cell pool by mediating the persistence of clones of low affinity for the activating antigen.

### Memory precursors of submaximal affinity express higher Bcl-2 protein levels

**Fig 4:**
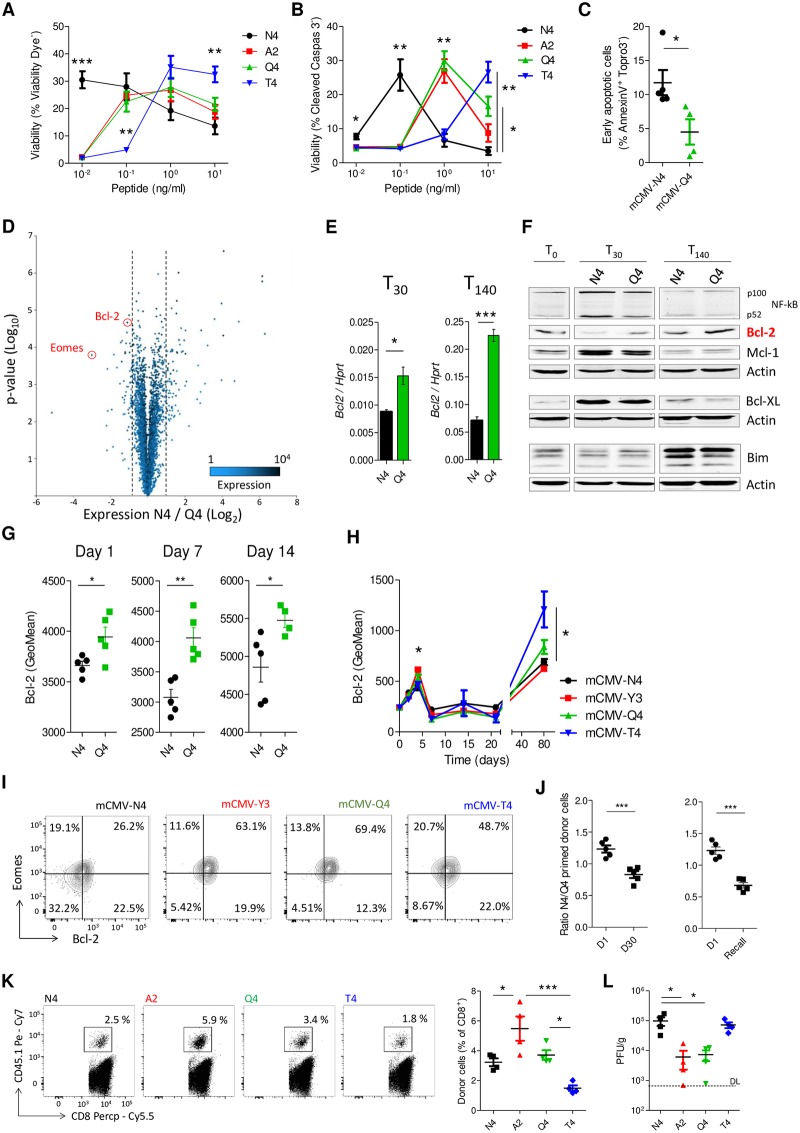
Memory precursors of suboptimal affinity express higher Bcl-2 protein levels. (**A,B**) Purified OT-1 cells were primed for 30 hours with indicated peptides and anti-CD28. Next, cells were washed and cultured for an additional 5 days with 50 ng/ml of IL-15. Shown is viability of cells on day 6 after stimulation primed with the indicated concentrations of peptides determined by staining for (**A**) viability dye or (**B**) cleaved caspase-3. (**C**) CD45.1^+^ OT-1 cells (10^4^) were transferred to WT CD45.2^+^ recipients. After 24 hours, mice were infected with mCMV-N4 or mCMV-Q4. Apoptosis was assessed on day 14 in donor cells from spleen by flow cytometry. (**D-G**) Purified OT-1 cells were primed for 30 hours with 1 ng/ml N4 or Q4 peptides and anti-CD28. Next, cells were washed and cultured for an additional 5 days with 50 ng/ml IL-15 to generate memory cells. (**D**) Differential expression was analyzed by microarray. Graph shows a volcano plot on day 6 of stimulation. Dashed lines indicate 2-fold differential expression. (**E-F**) Thirty (T30) and 140 (T140) hours after start of the experiment, cells were analyzed by (**E**) qPCR and (**F**) western blot. (**G**) On day 6 of stimulation, 1 × 10^6^ OT-1 cells (CD45.1^+^) were transferred to WT recipients (CD45.2^+^) and on the indicated time points after transfer expression of Bcl-2 was determined in donor cells in spleen by flow cytometry. (**H,I**) CD45.1^+^ OT-1 cells were transferred in WT (CD45.2^+^) recipients. After 24 hours, mice were infected with mCMV expressing the indicated peptides. (**H**) Quantification of the GeoMean of Bcl-2 in donor cells at the indicated days. See also S5E Fig. (**I**) Representative FACS plots show Eomes and Bcl-2 expression on day 4 after infection gated for CD8^+^CD45.1^+^ cells. (**J**) Memory OT-1 cells were generated in vitro after priming with N4 or Q4 peptides using CD45.1^+^ or CD45.1/2^+^ cells, respectively. On day 6 of stimulation, cells were mixed in equal proportions, and 1.4 × 10^6^ cells were transferred to WT recipients (CD45.2^+^). On days 1 and 30 after transfer, the ratio between donor cells in spleen was determined by flow cytometry (left panel). The same cell mixtures were transferred to WT recipients (CD45.2^+^) at 25,000 cells per recipient, and after 30 days, mice were infected with mCMV-N4. On days 1 and 37 after transfer, the ratio between donor cells in spleen was determined by flow cytometry (right panel). (**K-L**) Memory CD45.1^+^ OT-1 cells were generated in vitro using the indicated peptides for priming. On day 6 of stimulation, 25,000 viable OT-1 cells were transferred to naïve CD45.2^+^ recipients. After 47 days, mice were infected with mCMV-N4. (**K**) Donor-cell numbers were determined in spleen on day 5 after infection by flow cytometry. Representative FACS plots are gated for CD8^+^ cells. (**L**) Virus titers were determined in liver on day 3 after infection by plaque assay. Experiments show representative data from at least two (**E,G, J-L**), three (**F**), or four (**A-C,H,I**) independent experiments using 3–5 mice per group. (**D**) Data of microarray analysis of three biologically independent samples, each pooled from 3–4 mice. In (**A,B,H,K,L**), ANOVA followed by Bonferroni posttesting; in (**C,E,G,J**), Student *t* test; and in (**L**), Kruskal-Wallis test were used to analyze difference between groups. Shown are means ± s.e.m. *P < 0.05, **P < 0.01, ***P < 0.001. Values for each data point can be found in S1 Data. A2, SAINFEKL; DL, detection limit; FACS, fluorescence-activated cell sorting; GeoMean, geometric mean; IL, interleukin; mCMV, murine cytomegalovirus; N4, SIINFEKL; qPCR, quantitative polymerase chain reaction; PFU, plaque-forming units; Q4, SIIQFEKL; T4, SIITFEKL; WT, wild-type.

The dominance of a given clone in the CD8 memory pool is determined by the proliferation rate of its precursors and their ability to survive into the memory phase. To investigate how antigen affinity affects proliferation and survival of memory precursors, we first used our in vitro model for memory formation. Cells were CFSE labeled and stimulated with N4 peptide or APLs, and CFSE dilution as well as Ki67 staining was followed over time. Affinity positively correlated with proliferation, especially early after activation (S2A and S5A and S5B Figs), which is in line with what we had observed in vivo (Fig 2E). In contrast, we observed that on day 6 of stimulation, the highest viability of cells was obtained at relatively low concentrations of N4 peptide stimulation, beyond which survival was reduced (Fig 4A). This curve was shifted to higher peptide concentrations for cells of lower affinity, resulting in a survival advantage of these cells at high peptide avidity at memory time points (Fig 4A). Cell death was mediated through apoptosis, since dead cells stained positive for cleaved caspase 3 and annexin V (Fig 4B and S5C Fig). To see whether the survival advantage of cells primed with low-affinity ligands in vitro also persists in vivo, naïve OT-1 cells were transferred to WT recipients, which were subsequently infected with mCMV expressing N4 or Q4 peptide. Indeed, we observed reduced apoptosis of donor cells in mice infected with mCMV-Q4 compared to animals infected with mCMV-N4 early during memory formation (Fig 4C). Together, these findings suggest that despite an early proliferative disadvantage, cells of low affinity for the activating ligand are not negatively selected during memory formation because of a relative survival advantage.

We subsequently aimed to understand on a molecular level how the relative survival advantage of memory precursors primed with low-affinity ligands is mediated. Transcriptome analysis of cells stimulated with N4 or Q4 peptides on day 6 of culture in our in vitro memory formation model showed that the prosurvival gene *Bcl2* was significantly higher expressed in cells primed with low-affinity ligands, which we could confirm by qPCR (Fig 4D and 4E). Indeed, of all genes associated with apoptosis, according to the KEGG database, *Bcl2* was most highly expressed in Q4-stimulated cells (S5D Fig). Western blot analysis revealed that also on a protein level, low-affinity cells express more Bcl-2 at memory time points (Fig 4F). In contrast, Q4-stimulated cells did not express higher amounts of the prosurvival proteins Bcl-XL and Mcl-1 or lower amounts of the proapoptotic protein Bim compared to N4-primed cells. This suggests that Bcl-2 is the dominant molecule that promotes survival of memory precursors of low affinity for the activating ligand.

We used two different in vivo models to validate the impact of affinity on Bcl-2 expression in memory precursors. First, in vitro–generated memory cells (CD45.1^+^) were transferred into WT recipients (CD45.2^+^), and Bcl-2 expression was determined at the indicated time points. We found that also in vivo, cells primed with low-affinity ligands retained higher expression levels of Bcl-2 (Fig 4G). Second, OT-1 cells (CD45.1^+^) were transferred into WT recipients (CD45.2^+^), which were subsequently infected with mCMV expressing N4 or APLs. In all cells, we observed two peaks of Bcl-2 expression, one early after stimulation and one at late time points, which correlated closely with those of Eomes (Fig 1G and 1h, and S5E Fig). Importantly, also after in vivo priming, Bcl-2 and Eomes protein levels inversely correlated with antigen affinity (Fig 4H and 4I, and S5F Fig).

To demonstrate in vivo that memory precursor cells stimulated with submaximal affinity have a survival advantage, in vitro–generated memory OT-1 cells primed with N4 or Q4 peptides were mixed in a 1:1 ratio and transferred to WT recipients, and the ratio was determined after 1 day, 30 days or 7 days after reinfection with mCMV-Ova on day 30 after transfer. This revealed that the ratio shifted in favor of cells primed with ligands of suboptimal affinity both before and after recall (Fig 4J). Homing or homeostatic proliferation was not affected by the affinity of priming (S5G and S5H Fig), indicating that priming of T cells with submaximal affinity indeed provides a survival advantage. Finally, we aimed to demonstrate that affinity-dependent differences in survival of memory precursors have a functional impact on a per-cell basis. Equal numbers of in vitro–generated memory cells (CD45.1^+^) were transferred into naïve WT recipients (CD45.2^+^). After 47 days, mice were infected with mCMV-N4. Animals that had received cells primed with A2 or Q4 peptides showed increased donor-cell numbers on day 5 after infection compared to N4-primed cells, which corresponded with reduced viral titers in spleen (Fig 4K and 4L).

Thus, memory precursors primed with ligands of submaximal affinity express higher levels of Bcl-2 than cells primed with maximal or with low affinity, which corresponds with a relative survival advantage during memory formation.

### Eomes directly drives expression of Bcl-2

**Fig 5:**
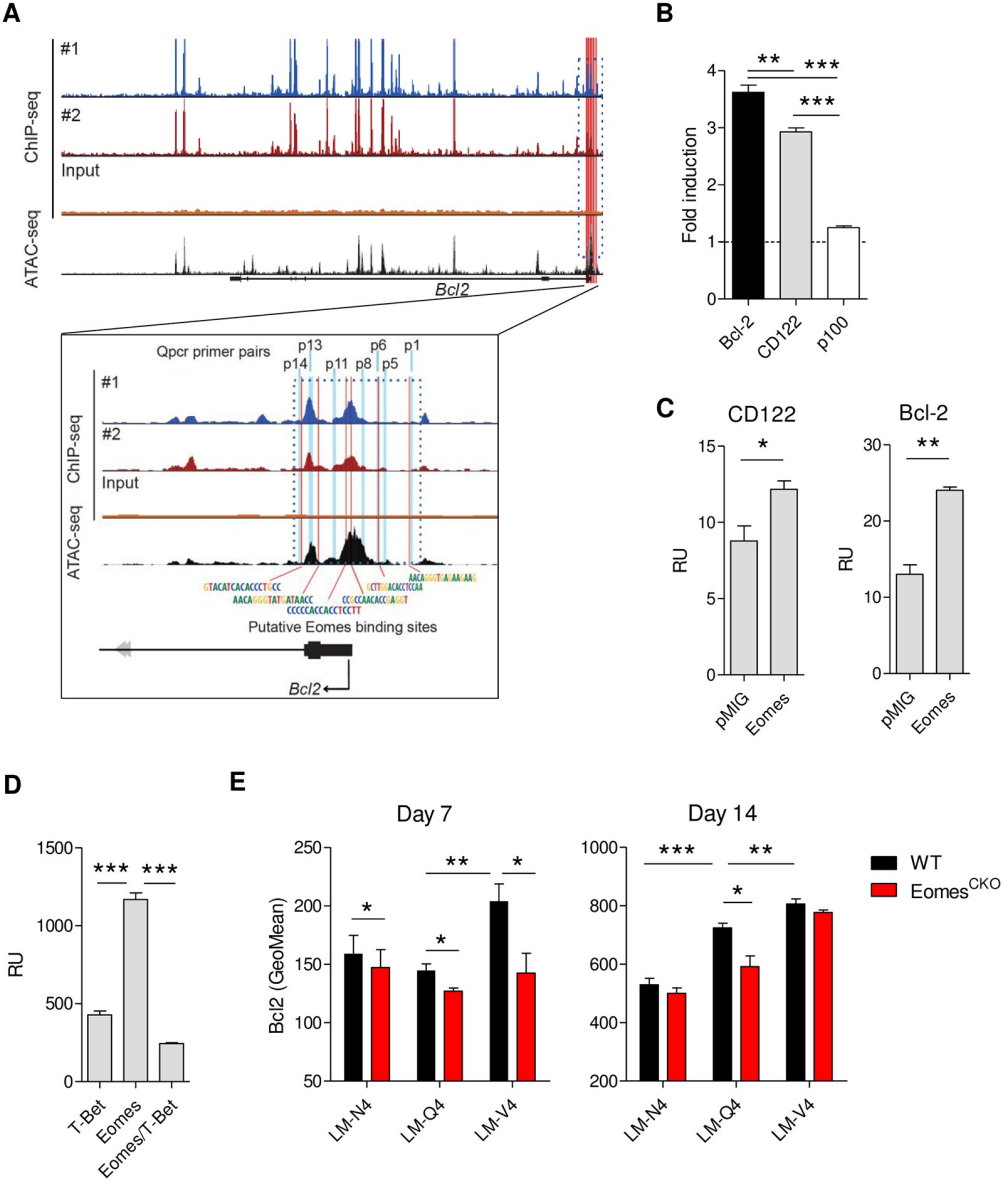
Eomes directly drives Bcl-2 expression. (**A**) OT-1 cells were stimulated for 30 hours with N4 peptide. Binding of Eomes to the *Bcl2* promoter was assessed by ChIP for two biological replicates and compared with ATAC-seq analysis. Light blue markers indicate sections amplified by qPCR (shown in S6C Fig). (**B**) Eomes^TG^ and empty vector (pMIG) containing NIH-3T3 cells were transfected with plasmids containing a luciferase gene preceded by the promoter of *Bcl2*, *Nfkb2*, or *Il2rb*. Cotransfection with a Tomato expression vector was used to normalize for transfection efficiency. Luminescence was analyzed 24 hours after transfection. Shown is fold induction over pMIG controls. (**C**) Eomes^TG^ HEK293 cells (Eomes) and empty vector–containing controls (pMIG) were transfected with plasmids containing a luciferase gene preceded by the promoter of *Bcl2* (Bcl2) or *Il2rb* (CD122). Cotransfection with a Tomato expression vector was used to normalize for transfection efficiency. Luminescence was analyzed 24 hours after transfection. Shown is signal intensity in RU after subtraction of background signal (mock-transfected samples). (**D**) T-bet^TG^ (T-bet), Eomes^TG^ (Eomes), and Eomes^TG^T-bet^TG^ (Eomes/T-bet) HEK293 cells were transfected with empty vector (mock) or a luciferase gene preceded by the promoter of *Bcl2*. Cotransfection with a Tomato expression vector was used to normalize for transfection efficiency. Luminescence was analyzed 24 hours after transfection. Shown is signal intensity in RU after subtraction of background signal (mock-transfected samples). (**E**) OT-1 (CD45.1/2^+^) and Eomes^CKO^ OT-1 (CD45.2^+^) cells were mixed in a 1:1 ratio, and 10,000 cells were transferred in WT (CD45.1^+^) recipients. Mice were infected with LM-N4, LM-Q4, or LM-V4. Expression of Bcl-2 was determined in donor-cell MPECs (CD127^+^KLRG1^-^) in the blood at indicated time points. (**A**) shows data from two biologically independent samples, each pooled from 3 mice. (**B-D**) are representative of three to nine experiments done in triplicate. (**E**) Shows representative plots of two experiments. In (**C,E**), Student *t* test was used, and in (**B,D**), ANOVA followed by Bonferroni posttesting was used to analyze difference between groups. Shown are means ± s.e.m. *P < 0.05, **P < 0.01, ***P < 0.001. Values for each data point can be found in S1 Data. ChIP-seq, chromatin immunoprecipitation sequencing; Eomes^CKO^, Eomes^flox/flox^ CD4Cre; LM, *L*. *monocytogenes*; MPEC, memory precursor effector cell; N4, SIINFEKL; Q4, SIIQFEKL; qPCR, quantitative polymerase chain reaction; RU, relative units; V4, SIIVFEKL; WT, wild-type.

Eomes expression has been associated with Bcl-2 [24,26], but direct transcriptional regulation has not been demonstrated. We therefore performed Eomes chromatin immunoprecipitation (ChIP) experiments using activated OT-1 cells. We observed strong binding of Eomes to the promoter regions of the known Eomes target gene *Prf1*, but not to *Ncr1*, which was included as a negative control (S6A and S6B Fig). In addition, Eomes bound to both the promoter and intronic regions of *Bcl2*, which we could confirm by qPCR. Moreover, peak intensities positively correlated with the amount of Eomes in the cell (Fig 5A and S6C Fig). Finally, we compared our Eomes ChIP sequencing (ChIP-seq) data with publicly available ATAC-seq data for activated CD8 T cells and found that Eomes binding closely corresponded with a subset of open chromatin regions within the *Bcl2* promoter (Fig 5A). Together, these data indicate that Eomes directly binds the promoter as well as intronic regions of *Bcl2*.

To confirm that Eomes not only binds the promotor region of *Bcl2* but also drives its expression, we generated NIH3T3 and HEK293 cell lines overexpressing Eomes and transfected them with plasmids containing a luciferase gene preceded by the promoter of *Bcl-2*, *Nfkb2*, or *Il2rb*. Ectopic expression of Eomes in NIH3T3 cells resulted in a strong induction of luciferase expression in cells transfected with the *Bcl2* promoter construct (Fig 5B). Expression was comparable or higher than cells transfected with a luciferase vector containing the promoter of *Il2rb* [27]. In contrast, expression of p100, included as negative control, was not induced by Eomes. Similar observations were obtained using human HEK293 cells (Fig 5C). Eomes and T-bet share the same DNA-binding motif [28]. Yet after high-affinity stimulation, when expression of both molecules is high, Bcl-2 levels are low. We therefore explored the possibility that T-bet represses Eomes-induced Bcl-2 expression. Indeed, HEK293 cells overexpressing both T-bet and Eomes had a much-reduced ability to drive luciferase expression by the Bcl-2 promoter compared to cells expressing only Eomes (Fig 5D).

Finally, we investigated whether Eomes is required for Bcl-2 expression of cells primed with low-affinity ligands in vivo. WT OT-1 (CD45.1/2^+^) and Eomes^CKO^ OT-1 (CD45.2^+^) cells were mixed in an equal ratio and transferred in WT recipients (CD45.1^+^). Recipient mice were infected with LM expressing N4 or APLs, and expression of Bcl-2 was determined in donor cells. Bcl-2 expression inversely correlated with antigen affinity in memory precursors. Importantly, Eomes^CKO^ cells showed lower levels of Bcl-2 early after low-affinity, but not high-affinity, priming compared to WT cells (Fig 5E). At late time points after infection, differences were mostly lost (S6D Fig).

Combined, these data indicate that Eomes directly drives Bcl-2 transcription in activated CD8 T cells, which is counteracted by T-bet.

### Eomes-mediated survival of low-affinity memory precursor depends on Bcl-2

Having confirmed that cells of low affinity express higher levels of Bcl-2, which is driven by Eomes, we investigated whether these cells also depend on Bcl-2 for their survival early after activation. OT-1 cells were stimulated in vitro with N4 or Q4 peptides and cultured in the presence of increasing amounts of ABT-199, a highly specific Bcl-2 inhibitor that is used in the clinics as an antileukemia drug under the name venetoclax [29]. Cells triggered with low-affinity or low-avidity peptides indeed depended more on Bcl-2 for their survival (S7A Fig). To analyze the impact of Bcl-2 inhibition on the overall affinity of antigen-specific CD8 T-cell populations in vivo, WT mice were infected with mCMV-N4 and, 3 or 6 days later, treated with the indicated dose of ABT-199. Analysis of antigen-specific memory precursors revealed a dose-dependent increase of antigen affinity following Bcl-2 inhibition for both days after ABT-199 injection, though the effect was stronger when it was administered on day 3 after infection (S7B and S7C Fig). These findings are in agreement with a model whereby low-affinity cells depend on Bcl-2 for survival.

**Fig 6:**
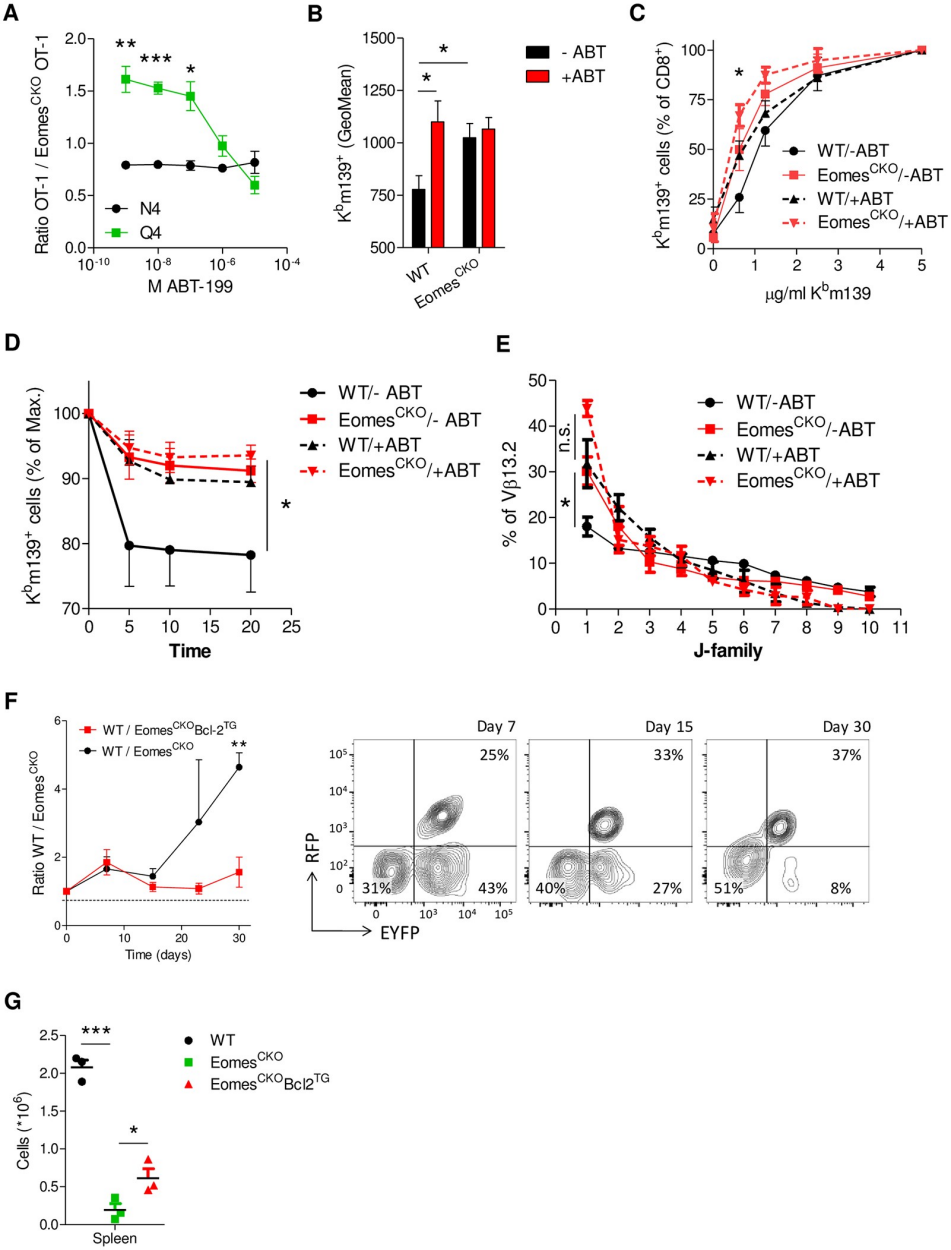
Eomes-mediated survival of low-affinity memory precursor depends on Bcl-2. (**A**) Purified OT-1 cells were cultured with the indicated peptides and anti-CD28 in the presence of increasing amounts of ABT-199. After 30 hours, viability was analyzed by flow cytometry. (**B-E**) mBMCs were generated using WT (CD45.1^+^) and Eomes^CKO^ (CD45.2^+^) cells in WT (CD45.1/2^+^) recipients. Eight weeks after reconstitution, animals were infected with mCMV-N4. Three days after infection, mice received a single injection i.p. with ABT-199 (+ABT) or carrier only (-ABT). (**B**) After 7 days, the GeoMean of K^b^m139^+^ and staining in spleen was analyzed by flow cytometry. (**C**) After 35 days, splenocytes were stained with increasing amounts of K^b^m139 tetramer, and the frequency of positive cells was analyzed by flow cytometry. Shown is the fraction of K^b^m139^+^ cells relative to staining with maximum concentration. (**D**) TCR:pMHC off-rates (tetramer decay) were determined in K^b^m139^+^ cells by flow cytometry. (**E**) mBMCs were infected with LCMV and injected with ABT-199 or carrier only (-ABT) after 6 days. After 30 days, WT and Eomes^CKO^ CD8^+^D^b^Gp33^+^ cells were sorted and analyzed by TCR sequencing. J-usage within Vβ13.2^+^ cells, ordered by abundance per clone, is shown. (**F,G**) Naïve EYFP^+^Eomes^CKO^ OT-1 (CD45.2^+^) cells were transduced with a lentiviral construct to overexpress Bcl-2 and RFP. RFP^+^EYFP^+^Eomes^CKO^ OT-1 cells were sorted, mixed in a 1:1:1 ratio with EYFP^+^Eomes^CKO^ OT-1 (CD45.2^+^) and OT-1 (CD45.1/2^+^) cells, and transferred to WT (CD45.1^+^) recipients. One day later, animals were infected with LM-Q4. (**F**) The ratio between donor OT-1 (WT) and EYFP^+^Eomes^CKO^ OT-1 (Eomes^CKO^) cells and between donor OT-1 and RFP^+^EYFP^+^Eomes^CKO^ OT-1 (Eomes^CKO^Bcl-2^TG^) donor cells was followed over time in the blood. FACS plots are gated for donor cells. Dashed line indicates ratio between total donor cells before injection in recipients. (**G**) Thirty days after primary infection, animals were reinfected with mCMV-N4. Seven days later, donor-cell populations were quantified in blood and spleen. Dotted line indicates initial frequency. In (**A,F**), Student *t* test was used, and in (**B-E,G**), ANOVA followed by Bonferroni posttesting was used to determine significant differences. Shown are representative graphs of two (**B-G**) or three (**A**) experiments using 3–5 mice per group. Shown are means ± s.e.m. *P < 0.05, **P < 0.01, ***P < 0.001. Values for each data point can be found in S1 Data. Eomes^CKO^, Eomes^flox/flox^ CD4Cre; EYFP, enhanced yellow fluorescent protein; FACS, fluorescence-activated cell sorting; GeoMean, geometric mean; i.p., intraperitoneally; LCMV, lymphocytic choriomeningitis virus; LM, *L*. *monocytogenes*; mBMC, mixed bone marrow chimera; mCMV, murine cytomegalovirus; N4, SIINFEKL; Q4, SIIQFEKL; RFP, red fluorescent protein; TCR, T-cell receptor; WT, wild-type.

To demonstrate in vitro that Eomes mediates survival of low-affinity cells through induction of Bcl-2, WT OT-1 (CD45.1^+^) and Eomes^CKO^ OT-1 (CD45.2^+^) cells were mixed in an equal ratio and cultured in the presence of increasing amounts of ABT-199 upon high- or low-affinity stimulation. Indeed, the survival advantage of WT cells upon low-affinity stimulation was negated by increasing amounts of ABT-199, whereas this ratio was unaffected upon TCR triggering with high-affinity ligands (Fig 6A). To confirm these findings in vivo, mBMCs were infected with mCMV-N4. After 3 days, animals were injected with 100 mg/kg ABT-199. Analysis of K^b^m139^+^ populations on day 7 after infection revealed that for both days 3 and 6 of Bcl-2 inhibition, an increase of the overall affinity of the WT cell pool was induced, which was not the case for Eomes^CKO^ cells (Fig 6B). Differences in affinity were retained into memory, since ABT-199 increased the signal in the WT but not the Eomes^CKO^ cell pool on day 41 after infection under conditions of limiting tetramer staining (Fig 6C). In addition, Eomes deficiency resulted in a reduced tetramer-dissociation rate compared to WT cells, which was negated by treatment of mBMCs with ABT-199 on day 3 after mCMV infection (Fig 6D). Similar observations were made when ABT-199 was injected on day 6 after infection (S7D and S7E Fig).

Next, we questioned whether Eomes requires Bcl-2 to allow for the persistence of low-frequency clones into the memory-cell pool. mBMCs were infected with LCMV and treated with ABT-199 after 6 days. Thirty days after infection, the clonal composition of WT and Eomes^CKO^ D^b^Gp33^+^ CD8^+^ T cells was determined within the dominant Vβ13.2^+^ family. We observed that ABT treatment resulted in a strong skewing of the J-usage within the antigen-specific Vβ13.2 populations (Fig 6E). Moreover, ABT-199 treatment resulted in a relative increase of highly expanded clones within the WT pool, causing differences between WT and Eomes^CKO^ cells to be lost (S7F Fig). Thus, Eomes-induced Bcl-2 mediates the persistence of low-frequency clones into the memory-cell pool.

Finally, we investigated in vivo whether lentiviral induced overexpression of Bcl-2 could prevent the loss of Eomes-deficient cells of low antigen affinity. Naïve OT-1^+^ (CD45.1/2^+^), EYFP^+^Eomes^CKO^ OT-1, and RFP^+^Bcl-2^TG^EYFP^+^Eomes^CKO^ OT-1 (Both CD45.2^+^) cells were mixed in equal ratios and transferred to WT (CD45.1^+^) recipients. Next, mice were infected with LM-Q4, and ratios were followed over time. As before, Eomes^CKO^ cells were progressively lost over time. Overexpression of Bcl-2 was able to prevent a relative reduction of these cells compared to WT OT-1 cells (Fig 6F). When mice were reinfected with mCMV-N4 at memory time points, Eomes^CKO^ OT-1 cells gave a strongly reduced recall response compared to WT OT-1 cell, which was partially prevented by overexpression of Bcl-2 (Fig 6G). These findings indicate that Bcl-2 pays an important, though possibly not exclusive, role in mediating survival of cells primed with submaximal affinity. Notably, after recall, Eomes-deficient cells were not impaired in their ability to produce cytokines upon restimulation, and this was not affected by overexpression of Bcl-2 (S7G and S7H Fig).

Together, these findings indicate that cells primed with ligands of low affinity require Eomes-driven Bcl-2 expression early after activation for survival and persistence into memory.

### The Eomes/Bcl-2 axis promotes the ability of the memory pool to respond to mutated pathogens

**Fig 7:**
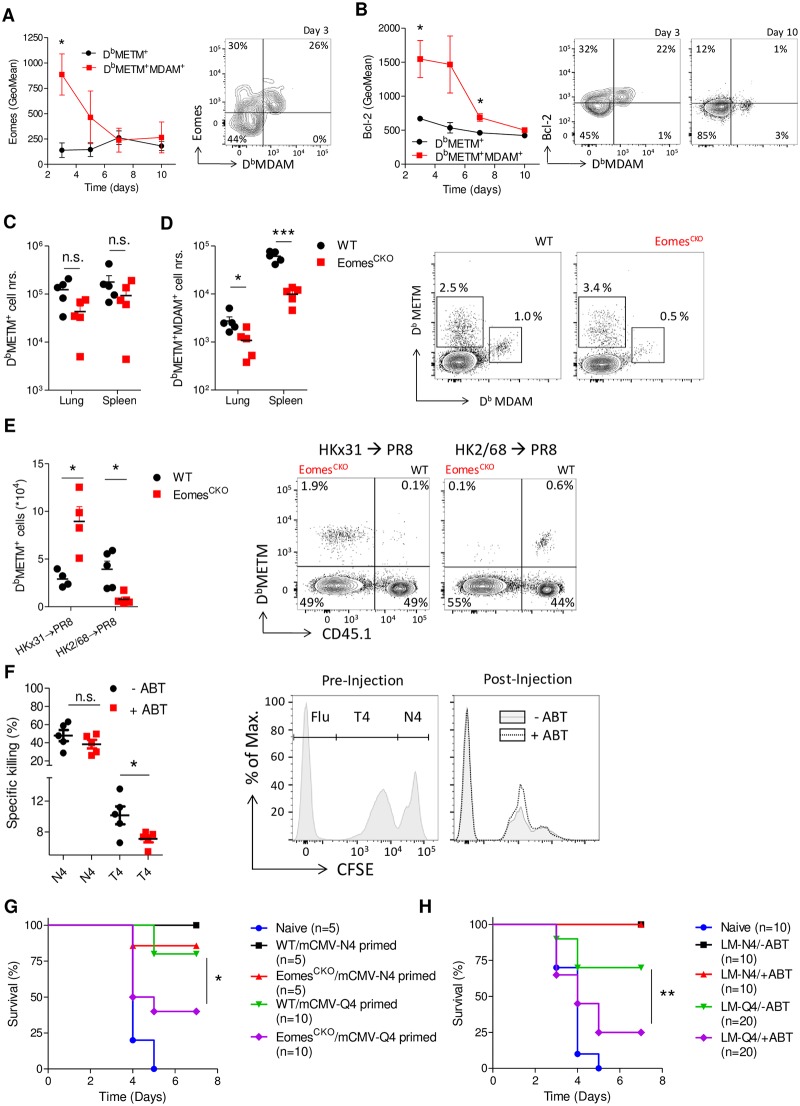
The Eomes/Bcl-2 axis promotes the ability of the memory pool to respond to mutated pathogens. (**A,B**) WT mice were infected intranasally with PR8, which carries the immunodominant epitope METM. At the indicated time points (**A**) Eomes and (**B**) Bcl-2 expression was quantified in D^b^METM^+^ and D^b^METM^+^D^b^MDAM^+^ CD8 T cells of the lung-draining lymph node. (**C,D**) mBMCs were infected intranasally with PR8. After 10 days, absolute numbers (“nrs.”) of (**C**) D^b^METM^+^ and (**D**) D^b^METM^+^D^b^MDAM^+^ cells were determined by flow cytometry. Representative plots show donor WT (left panel) and Eomes^CKO^ (right panel) CD8 T cells from the same animal in spleen. (**E**) mBMCs were infected with influenza A strain HKx31 (METM^+^) or HK2/68 (MDAM^+^). After 35 days, mice were reinfected with PR8 (METM^+^), and recall responses were determined after 10 days. Representative FACS plot shows donor CD8^+^ cells in spleen. (**F**) WT CD45.2^+^ mice were infected with mCMV. After 6 days, animals received ABT-199 (+ABT) or carrier only (-ABT). On day 35, mice were injected with CFSE-labeled CD45.1^+^ splenocytes, pulsed with control, N4, or T4 peptides. Specific killing of N4- or T4-pulsed target cells relative to control-pulsed control cells was determined in spleen by flow cytometry. Representative FACS plots for CFSE-stained populations pre- and post injection. Gated is for donor CD45.1^+^ cells. (**G**) WT OT-1 (WT) or Eomes^CKO^ OT-1 (Eomes^CKO^) cells (10^4^) were transferred to WT recipients and primed with mCMV-N4 or mCMV-Q4. After 30 days, mice were infected with a lethal dose of LM-N4 and survival was followed. Nonprimed (Naïve) mice were included as a control. (**H**) WT mice were infected with LCMV-N4 and, after 3 days, treated with ABT-199 or carrier only (-ABT). After 30 days, mice were infected with a lethal dose of LM-N4 or LM-Q4 and survival was followed. Naïve mice were included as a control. In (**A-F**), Student *t* test was used, and in (**G,H**), log-rank test was used to determine significant differences between groups. Shown are representative graphs of two (**C-G**) or three (**A,B**) experiments using 4–10 mice per group. (**H**) shows pooled data from two independent experiments. Shown are means ± s.e.m. *P < 0.05, **P < 0.01, ***P < 0.001. Values for each data point can be found in S1 Data. CFSE, carboxyfluorescein succinimidyl ester; Eomes^CKO^, Eomes^flox/flox^ CD4Cre; FACS, fluorescence-activated cell sorting; GeoMean, geometric mean; LCMV, lymphocytic choriomeningitis virus; LM, *L*. *monocytogenes*; mBMC, mixed bone marrow chimera; mCMV, murine cytomegalovirus; MDAM, ASNENMDAM; METM, ASNENMETM; N4, SIINFEKL; n.s., not significant; PR8, influenza A strain PR/8/34; Q4, SIIQFEKL; T4, SIITFEKL; WT, wild-type.

The clinical relevance from a clonally diverse memory pool comes from its increased scope and therefore enhanced ability to respond to pathogens carrying mutations compared to the original pathogen [4]. We therefore investigated the ability of Eomes-deficient memory cells to respond to reinfection with mutated pathogens. First, we aimed to confirm affinity-mediated regulation of Eomes and Bcl-2 early after CD8 T-cell activation, using a model in which we could directly compare clones of low and high affinity directed against the same antigen. Mice infected with PR8 generate a dominant response against the viral epitope ASNENMETM (METM), but a minor fraction of cells is also able to bind the APL ASNENMDAM (MDAM). These cells are derived of different clones and have a lower affinity for D^b^METM than cells without cross-reactive capacity [4]. Indeed, we observed that after PR8 infection, cross-reactive cells express higher levels of both Eomes and Bcl-2 than cells that only bound D^b^METM early during the immune response (Fig 7A and 7B). Moreover, the kinetics of expression closely correlated between these two molecules. Differences in expression of Eomes and Bcl-2 between the two populations were lost at later time points (Fig 7A and 7B).

Next, mBMCs were infected with PR8. Early after infection, the absolute number of D^b^METM^+^ cells was not reduced in absence of Eomes (Fig 7C). In addition, overall affinity was increased in the antigen-specific Eomes^CKO^ cell pool (S8A Fig). Importantly, we observed a reduction in the number of cells able to bind both D^b^MDAM and D^b^METM already early after infection, both in spleen and lung (Fig 7D). To investigate whether Eomes deficiency impairs cross-reactive memory responses, mBMCs were infected with influenza HKx31, which expresses the METM epitope, or with HK2/68, which expresses MDAM. Antigen-specific CD8 T cells were followed over time in blood. We observed that the WT CD8 T-cell pool formed significantly more D^b^MDAM^+^D^b^METM^+^ cross-reactive cells than cells lacking Eomes. WT cells also obtained a memory phenotype more quickly (S8B and S8C Fig). After 30 days, mice were reinfected with PR8 (METM), and recall responses were analyzed 10 days later. We observed that Eomes-deficient cells gave a better recall response against pathogens carrying the original epitope (HKx31→PR8). In contrast, Eomes^CKO^ cells demonstrated a strongly reduced recall response against virus carrying a “mutated” epitope ([HK2/68→PR8], Fig 7E). Thus, Eomes mediates sufficient diversity of the memory pool in order to increase its scope and ensure reactivity against pathogens with mutations in their immunodominant epitopes.

To investigate whether we can alter the scope of the cytotoxic CD8 T-cell response by pharmacological targeting of the Eomes/Bcl-2 axis during priming, we infected WT mice with mCMV-N4 and, after 6 days, treated them with ABT-199. On day 35 after infection, mice were injected with CFSE-labeled splenocytes pulsed with control, N4, or T4 peptides, and specific killing was determined in the spleen. Inhibition of Bcl-2 had no impact in killing of cells pulsed with the original antigen but caused a significant reduction in their ability to kill “mutated,” low-affinity targets (Fig 7F).

Finally, we aimed to demonstrate that by targeting the Eomes/Bcl-2 axis, the CD8 memory T-cell pool has a reduced capacity to protect mice from lethal reinfection with “mutated” pathogens. First, WT or Eomes^CKO^ OT-1 T cells were transferred to WT recipients, which were then infected with mCMV-N4 or mCMV-Q4. After 30 days, animals were infected with a dose of LM-N4 that is lethal for naïve mice. We observed that all mice primed with mCMV-N4 survived LM-N4 infection, irrespective of the genotype of donor cells. In contrast, in groups primed with mCMV-Q4, significantly more mice transferred with Eomes^CKO^ OT-1 cells died than animals that had received WT OT-1 cells (Fig 7G). To demonstrate that this effect was mediated through Bcl-2, WT mice were infected with LCMV-N4 and injected with ABT-199 or vector control 3 days later. One month after priming, animals were infected with a dose of LM-N4 or LM-Q4 that is lethal for naïve mice. We observed that ABT-199 did not diminish the protective capacity of cells challenged with pathogens expressing the original ligands. In contrast, following challenge with a pathogen carrying a “mutated” ligand, significantly more mice died in the group treated with ABT-199 (Fig 7H).

Thus, the Eomes/Bcl-2 axis plays an important role in maintaining clonal diversity within the memory CD8 T-cell pool, which increases the number of pathogenic variants against which it can provide protection upon reinfection.

## Discussion

The scope of the CD8 memory T-cell pool depends on its clonal composition and determines its ability to cope with reinfection of both the original pathogen and its mutant forms [30]. Various groups have investigated the impact of antigen affinity on effector-cell formation [9,15,16,31,32] or on the ability of low-affinity cells to form memory [9,17], mostly in the context of monogenic TCR transgenic systems. How low- and high-affinity cells compete for the memory niche has been largely neglected. We find that memory precursor survival is optimized within a window of TCR stimulation early during priming, confined by the affinity and avidity of the trigger. Cells of submaximal affinity therefore have an intrinsic survival advantage, which enables them to compete for the memory niche. Cells of very low affinity fail to induce sufficient amounts of prosurvival proteins and are therefore negatively selected. High-affinity cells, in contrast, proliferate faster, which ensures their dominant contribution to the memory pool, despite a survival disadvantage. In absence of Eomes or when Bcl-2 is inhibited, only high-affinity cells can form memory. Thus, a dynamic balance between a limited number of transcriptional regulators determines the scope of the memory pool.

The effector CD8 T-cell pool is stringently selected for clones with the highest antigen affinity, and this process has been well studied [3,33]. Effector-cell selection is mediated through increased proliferative capacity of high-affinity clones [9] and through death of low-affinity clones because of a reduced ability to interact with dendritic cells [34], leading to a shorter retention time in the lymph node [32] and an inability to sustain sufficient levels of the prosurvival protein Mcl-1 [2,15]. Whereas low-affinity memory precursors also have lower levels of Mcl-1 than high-affinity cells, we find that this selection mechanism is “cheated” during memory formation through direct induction of Bcl-2 by Eomes. This process promoted responsiveness of the memory pool to target cells carrying APLs, a model for reinfection with pathogens carrying point mutations in immunodominant epitopes. Increased clonal diversity only marginally impacted recall responses against high-affinity ligands, indicating that relaxed selection for affinity is an acceptable strategy for memory formation.

Eomes is a homologue of T-bet with an identical DNA-recognition motif [35]. Originally, Eomes was therefore identified as a mediator of effector differentiation, as its overexpression was shown to induce Granzyme B expression in activated T cells [35,36]. Other studies found a strong correlation between Eomes expression and memory potential, which could be suppressed by strong proinflammatory stimuli such as IL-2 and IL-12 [37–40]. Models using exclusively the TCR transgenic OT-1 model indicated that Eomes expression is higher in cells activated with low-affinity ligands [16,17]. How all these observations could be integrated in a general model of effector and memory formation remained elusive. Our findings indicate that the primary role of Eomes-dependent memory formation is to ensure a broad scope of the memory pool. Whereas expression of Eomes and of its direct targets *Il2rb* and *Bcl2* follows an “optimum curve,” T-bet levels are proportional to TCR affinity [13]. T-bet is induced at higher cumulative activating signal intensity and suppresses expression of Eomes [39] and of Bcl-2. In effector cells, Eomes is therefore redundant, apart from a minor role in induction of KLRG1, but can substitute for loss of T-bet [23]. In memory-cell formation, Eomes has mostly been studied using overexpression models. Under those conditions, Eomes mediates the prosurvival role that it normally only executes in cells activated with low-affinity ligands also in cells of high affinity [26,41,42]. This explains why a much broader role has been attributed to Eomes in memory formation than appears to be the case on a physiological level.

The model presented here may also provide better understanding of the formation of nonconventional memory CD8 T-cell populations. Virtual memory cells (VMs) occur in the absence of an overt pathogenic trigger and are characterized by high expression of Eomes and Bcl-2 [43–45]. They can be found in naïve mice and postnatal infants [43,46] but are exceptionally expanded under conditions of lymphopenia [47]. Notably, VM formation was shown to depend on the induction of Eomes via low-affinity interaction with self-peptides [47,48]. Conversely, tissue-resident memory T cells (T_RM_), which are noncirculating memory CD8 T cells that reside in peripheral tissues, were shown to be of particularly high affinity for the original antigen and strongly down-regulate expression of Eomes [49,50]. Whereas we did not directly investigate these populations, it is tempting to speculate that TCR affinity–mediated regulation of Eomes plays a role also in the formation of these memory CD8 T-cell subsets.

Our findings shed valuable new light on the molecular mechanism of memory CD8 T-cell formation. We show that cells activated with low-affinity ligands “cheat” the system in their competitive struggle with high-affinity cells for prosurvival factors by adopting a superior cell-intrinsic survival capacity. This process ensures sufficient diversity of the memory-cell pool. Importantly, we demonstrate that manipulation of TCR signaling provides an attractive target for the development of CD8 T-cell vaccines with an altered scope.

## Materials and methods

### Ethics statement

All animal experiments were performed after approval by the Dier Ethische Commissie AMC Amsterdam (approval no. DSK56) and the Uprava za veterniarstvo I sigurnost hrane, Odjel za zaštitu životinja (approval no. 525-10/0255-17-4). Animal experiments were performed in accordance with the European Union directive 2010/63/EU.

### Mice

Mice were strictly age- and sex-matched within experiments and handled in accordance with institutional and national guidelines. B6 (line 664), OT-1 (3831), Eomes^fl/fl^ (17293), and B6 CD45.1 (2014) mice were purchased from the Jackson Laboratory. CD4^cre^ mice were provided by D. Littman (New York, NY, United States of America). Rosa^YFP^ mice were provided by A. Waisman (Mainz, Germany). Mx1^Cre^ mice were provided by K. Rajewsky (Cologne, Germany). All lines were kept as breeding colonies in our local animal facility under specific pathogen–free conditions. All lines were either generated on a B6 background or backcrossed at least 12 times on this background.

### In vitro stimulations

CD8 T cells were purified by positive selection using magnetic beads (Miltenyi Biotec). Cells were cultured in RPMI 1640 medium (PAN-Biotech), supplemented with 10% FCS (PAN-Biotech) and 2-ME (Sigma-Aldrich). For in vitro memory differentiation, 30,000 cells per well in a U-bottom 96-well plate (Cellstar) were stimulated for 30 hours with APLs (Genscript), washed, and cultured for an additional 5 days with 50 ng/ml IL-15 or 100 ng/ml IL-2 (PreproTech).

### Surface and intracellular staining, antibodies, and tetramers

All antibodies were purchased from eBioscience and BD Biosciences. MHC class I tetramers were generated in-house (Sanquin Research). CFSE, annexin V (eBioscience), and BrdU (BD Biosciences) labeling and staining were performed according to the manufacturers’ protocols, preceded by blocking of Fc receptors using 2.4G2 antibodies (in-house generated). For BrdU labeling, mice were injected i.p. with BrdU (1 mg) in 500 μL of phosphate buffered saline (PBS; PAN-Biotech). BrdU incorporation in CD8 T cells was visualized using the BrdU flow kit (BD Biosciences). Fixation and permeabilization of cells for intracellular stainings (IFNγ, TNF, IL-2, Ki67 eBioscience; Cleaved caspase 3, Cell Signaling) were performed using the BD Fix/Perm kit. For intranuclear staining (T-bet, Eomes, Bcl-2), the eBioscience Fix/Perm kit was used. For cytokine staining, cells were first restimulated for 4 hours in vitro with 10 ng/ml peptides (Gp33; SCLEFWQRV [M57]; TVYGFCLL [m139]; N4) (Genscript) in the presence of brefeldin A (eBioscience). Flow cytometric analysis was done on a FACSVerse and FACSCanto (BD Biosciences), and sorting was performed on FACSAria (BD Biosciences) and analyzed with FlowJo software (Tree Star).

### TCR dissociation assay

mBMCs were infected with mCMV infection, and after 30 days, splenocytes were stained with anti-CD8 and K^b^m139 tetramer for 1 hour at room temperature. Cells were washed and incubated in the presence of saturating amounts of anti-H-2Kb antibody (AF6-88.5.5.3) at room temperature to prevent rebinding. At the indicated times, cells were washed and fixed, and the amount of remaining K^b^m139 tetramer on the surface was quantified by flow cytometry. Values are presented as a percentage of maximum staining at T = 0.

### Virus and bacterial infections and tumor model

The bacterial artificial chromosome (BAC)-derived mCMV strain MW97.01 (in-house produced) has previously been shown to be biologically equivalent to the mCMV Smith strain (VR-1399; ATCC) and is referred to as mCMV. mCMV-N4 was generated as described [51]. The recombinant mCMV-APLs mCMV-Y3, mCMV-Q4, and mCMV-T4 were generated by two-step replacement BAC mutagenesis, analogue to mCMV-N4, by orthotopic peptide swap [52] in the mCMV BAC plasmid pSM3fr [53], using the shuttle plasmids pST76K-m164_SIYNFEKL, pST76K-m164_SIIQFEKL, and pST76K-m164_SIITFEKL. The recombinants mCMV-N4-Δm157 and mCMV-N4-Δm152 were generated from pSM3frΔm152 [54] and pSM3frΔm157 [55] by using pST76K-m164_SIINFEKL [51]. All mCMVs were propagated on mouse embryonic fibroblasts (MEFs) and purified by standard protocol. Adult mice (6–12 weeks old) were infected IV with 2 × 10^5^ PFU of the indicated mCMV, and virus titers were determined on MEFs by standard plaque assay. LCMV Armstrong strain (Armstrong E-350; ATCC) and LCMV-N4 [56] was propagated on baby mouse kidney cells according to standard protocol. Animals were infected i.p. with 10^6^ PFU. For infection with LM overexpressing APLs (N4/Q4/V4) [9] or Gp33 [57], bacteria were cultured to an exponential growth phase. For priming or analysis of recall responses, mice were injected IV with 2.5 × 10^3^ CFU per mouse. For survival experiments, animals were injected with 10 × 10^4^ CFU per mouse. The H1N1 PR8 and H3N2 strains HKx31 and HK2/68 [4] were generated in LLC-MK2 cells, and TCID_50_ was determined in WT B6 mice. Mice were infected intranasally with 10 × TCID_50_ under ketamine/xylazine anesthesia. The B16-N4 cell line was provided by Tim Sparwasser (TWINCORE, Hannover). Cells were cultured in 10% DMEM (Lonza) supplemented with β-mercaptoethanol (PAN-Biotech) and under G418 selection (InvivoGen). Mice were rechallenged by 105 B16-N4 cells IV. Prior to quantification of metastases, lungs were bleached in Fekete solution.

### In vivo experiments

Bone marrow (BM) recipients were lethally irradiated (9 Gy). Recipient mice received 10^7^ donor BM cells IV. mBMCs received WT and Eomes^CKO^ BM mixed in a 1:1 ratio. Experiments were performed at least 8 weeks after transfer. OT-1, Eomes^CKO^ OT-1, and Eomes^iCKO^OT-1 cells were transferred IV. For adoptive transfer, CD8 T cells were purified via magnetic separation according to the manufacturers’ protocols (Miltenyi Biotec). mBMCs were treated with ABT-199 (MCE) by one i.p. injection of 25–100 mg/kg in 500 μL of sunflower seed oil (Sigma-Aldrich). For activation of Cre recombinase in Eomes^iCKO^ cells, 400 μg per mouse of Poly(I:C) (Sigma-Aldrich) in 500 μL of PBS (PAN-Biotech) was injected i.p. For in vivo killing, mice were infected with mCMV. After 6 days, animals received 100 mg/kg ABT-199 IV. After 7 and 35 days, animals were injected IV with 10^7^ CD45.1 splenocytes. Before transfer, splenocytes were pulsed with N4, T4, or PB1-F2 (Flu) peptides and mixed in a 1:1:1 ratio. Cells could be distinguished based on differential CFSE (Molecular Probes) labeling. On day 7 at 4 hours and on day 35 at 16 hours after transfer, specific killing of APL-pulsed cells, relative to Flu-pulsed cells, was determined in spleen using flow cytometry. For labeling of red pulp CD8 T cells, mice were injected with CFSE (25 nmol), and after 5 minutes, mice were humanely killed and perfused with PBS. Red pulp cells were defined as CFSE^+^ by flow cytometry.

### Lentiviral transduction

Naïve EYFP^+^Eomes^CKO^ OT-1 (CD45.2^+^) CD8 T cells were transduced with a lentiviral construct to overexpress Bcl-2 and RFP (Amsbio; LVP553) according to manufacturers’ protocol. RFP^+^EYFP^+^Eomes^CKO^ OT-1 cells were sorted using FACSAria sorter and mixed in a 1:1:1 ratio with EYFP^+^Eomes^CKO^ OT-1 CD8 T cells (CD45.2^+^) and OT-1 CD8 T (CD45.1/2^+^) cells, and 200 cells were transferred to WT (CD45.1^+^) recipients. One day later, animals were infected with LM-Q4. Thirty days after primary infection, animals were reinfected with mCMV-N4. Donor-cell populations were quantified in blood and spleen over time using flow cytometry.

### Western blot and qPCR

Samples for western blot were lysed in Laemmli lysis buffer (0.12 M Tris HCL [pH 8], 4% SDS, 20% glycerol, 0.05 μg/μL bromophenol blue, and 50 mM dithiothreitol) and boiled for 5 min. Protein contents were determined by the Bio-Rad protein assay (Bio-Rad Laboratories), and equal amounts of total lysate were analyzed by 12% SDS–polyacrylamide gel electrophoresis. Proteins were transferred to Immobilon-P and incubated with blocking buffer (Tris buffered saline/Tween-20 [TBS-T]) containing 2% low-fat milk for 1 hour before incubating with an antibody against Mcl-1 (BD Pharmingen), Bcl-XL (Transduction Laboratories), Bim (Stressgen Bioreagents), Bcl-2 (Enzo Lifesciences), p100/p52 (Cell Signaling), or β-actin (Santa Cruz Biotechnology) overnight at 4 °C in TBS-T. Blots were subsequently incubated with IRDye 680 or 800 labeled secondary antibodies (Li-Cor) for 1 hour. Odyssey Imager (Li-Cor) was used as a detection method according to the manufacturers’ protocols. For RT-PCR, total RNA was extracted using the TRIzol isolation method (Invitrogen). cDNA was generated using the SuperScript II cDNA synthesis kit (Invitrogen). Transcripts were amplified by PCR using an ABI 7500 RT-PCR system. We used *Hprt* as a housekeeping gene for normalization.

### Analysis of TCR clones

For the analysis of influenza-specific clones, WT mice were infected with PR8. On days 10 and 87 after infection, mice were humanely killed and splenocytes isolated. Total RNA was extracted from CD3^+^CD4^-^CD8^+^D^b^NP366^+^ sorted T cells using the TRIzol (Invitrogen) isolation method, followed by purification on RNeasy columns (Quiagen) and sodium acetate purification. For TCR sequencing, cDNA was generated using SuperscriptII-RT and oligo-dT according to the manufacturers’ protocols (Invitrogen #18064). The complementarity determining region 3 (CDR3) of the β-chain was used as a unique tag for clonal expansions. cDNA was amplified using a primer specific for the Vβ8.3 gene segment and a primer specific for the Constant segment. Amplification products were purified using AMPure SPRI beads (Agencourt Bioscience). The primers were tailed with the primerA and primerB sequences that allow high-throughput sequencing using Genome Sequencer FLX (Roche Diagnostics) and identification of the CDR3 regions as described previously [58]. Preparation of the samples and sequencing was performed according to the manufacturers’ protocols.

For the analysis of LCMV-specific clones, mBMCs containing WT and Eomes^CKO^ cells in an equal ratio were infected with LCMV. After 6 days, animals were injected with 100 mg/kg ABT-199. On day 30, total RNA was extracted from CKO (CD45.2^+^ EYFP^+^) and WT (CD45.1^+^) CD8^+^D^b^Gp33^+^ sorted T cells. Collected murine samples were lysed in 350 μL Buffer RLT (Qiagen) plus 1% β-mercaptoethanol (Sigma-Aldrich) and stored at −80 °C until RNA isolation, performed with RNeasy Mini Kit (Qiagen) according to the manufacturer’s instructions. RNA was quantified at NanoDrop 2000/2000c Spectrophotometer (Thermo Fisher Scientific). RNA (250 ng) was used for cDNA synthesis with custom primers containing a specific sequence for TCRβ C region (adapted from [59]), a Unique Molecular Identifier (UMI) [60], and the Nextera Read 1 adapter (Illumina). cDNA synthesis was performed using SuperScript III Reverse Transcriptase (Thermo Fisher Scientific) according to manufacturer’s instructions for custom primers procedure. cDNA is purified to remove leftovers primers using Agencourt AMPure XP beads (Beckman Coulter) using 1:1 ratio. Purified cDNA (125 ng) was used for PCR using Nextera Index 1 Read reverse primers and a multiplex of forward primers containing TCRβ V genes sequences (adapted from [59]) and Nextera Read 2 adapter. PCR was performed using HOT FIREPol DNA Polymerase (Solis BioDyne) as follows: 96 °C 15 minutes, 40× (96 °C for 30 seconds, 60 °C for 60 seconds, 72 °C for 30 seconds), 72 °C for 10 minutes. Obtained PCR products were purified using Agencourt AMPure XP beads using a 1:1 ratio and further processed for next-generation sequencing (NGS) as described below. Purified PCR products were processed for NGS using MiSeq Reagent Kit v3 600 cycles (Illumina) according to the manufacturer’s instructions. Sequencing was performed on the Illumina MiSeq platform. On average, 21,944 (±21,667) reads were obtained for all samples. TCRβ clones were identified according to their unique V-CDR3-J combination via a customized bioinformatics pipeline as described earlier [58].

### Microarray analysis

For microarray analysis, 100 ng total RNA was amplified using the GeneChip 3' IVT Plus Reagent kit (Thermo Fisher Scientific) generating biotinylated complementary RNA. The labeled samples were hybridized to GeneChip HT MG-430 PM arrays (Thermo Fisher Scientific). Washing, staining, and scanning was performed using the GeneTitan Wash, Stain Kit for 3' IVT Array Plates, and the GeneTitan Instrument (Thermo Fisher Scientific). Data were analyzed using the R2 Genomics analysis and visualization platform (http://r2.amc.nl).

### Generation of transgenic cells and luciferase assay

NIH-3T3 and HEK293T cell lines were purchased (ATCC). Cells were cultured in IMDM (PAN-Biotech), supplemented with 10 mM HEPES (pH 7.2), 2 mM L-glutamine, 10^5^ U/L Penicillin, 0.1 g/L Streptomycin, and 10% FCS. Cells were maintained in an incubator at 37 °C, 5% CO_2_. In order to generate retroviral particles, Phoenix-Eco and Phoenix-Ampho cells (ATCC) were transfected with retroviral vectors MSCV-Eomes-IRES-GFP and MSCV-IRES-GFP (provided by Professor Gabrielle Belz [WEHI]) using a GENIUS DNA Transfection Reagent (Westburg), following the manufacturers’ protocols. Transduction of NIH-3T3 and HEK293T cells was performed by 1 cycle of overnight exposure to viral supernatant (containing mock vector or Eomes-IRES-GFP). GFP-positive cells were sorted on a FACS sorter (Moflow; Dako-Cytomation, Carpinteria, CA, USA). For T-bet overexpression, HEK293T Eomes^TG^ cells were transfected with a T-bet expression vector (GenScript; OHu19893) using GENIUS transfection reagent. Cells were selected using G418 (Sigma-Aldrich). For luciferase assays, we first transfected transgenic cells with plasmids containing a luciferase gene preceded by the promoter of *Bcl-2* (AddGene #15381), or *Nfkb2* or *Il2rb* promoter regions, which were amplified by PCR. A 1-kb region upstream of human p100 translation start was amplified using the following primers: 5′-AGAGGTTGCAGTGAGCCAAAATCC-3′ and 5′-GGTGTGGGTGCAGGCACC-3′. The 1.5-kb upstream promoter region of murine *Il2rb* was amplified using the following primers: 5′-GTTTGAGTGCCTTTTCTTAGG-3′ and 5′-CAGCTCCTCTCAGCTGTG-3′. Either fragment was subcloned into pGL3 upstream of a gene encoding luciferase. Cells were transfected with the resulting construct together with a plasmid encoding dTomato (Addgene #30530) using GENIUS transfection reagent. Transfection efficiency was determined after 24 hours by measuring the percentage of Tomato (PE)-positive cells by flow cytometry. After 48 hours, luciferase activity was determined by aspiration of the medium followed by addition of luciferase reagent (0.8 mM ATP, 0.8 mM D-luciferin, 18 mM MgCl2, 0.8 mM Na2H2P2O7, 39 mM Tris-H_3_PO_4_ [pH 7.8], 0.4% glycerol, 0.03% Triton-X100, and 2.6 μM dithiothreitol). Luminescence was measured in a Synergy HT (Biotek, Winooski, VT, USA).

### Quantification and statistical analysis

Unless otherwise noted, data are presented as mean ± SEM. Statistical significance was determined by either Student *t* test or one-way ANOVA with Bonferroni posttesting. Differences in viral titers between experimental groups were determined by Kruskal-Wallis test, followed by Dunn posttesting. Statistical analysis was performed using GraphPad Prism 5 (GraphPad Software). *P* values of <0.05 (*P < 0.05, **P < 0.01, and ***P < 0.001) were considered statistically significant.

### ChIP-seq analysis

CD8 T cells (10^7^) from OT-1 mice were used for each ChIP-seq sample, adapting published protocols [61]. Briefly, CD8+ T cells were spun down and fixed in 1 mg/ml DSG (Thermo Fisher Scientific) in PBS for 30 minutes at room temperature, followed by addition of 1% formaldehyde and an additional 10-minute fixation at room temperature. The cross-link reaction was quenched with 0.125 M glycine, washed three times in PBS, and snap frozen and stored in −80 °C for ChIP. Nuclei were isolated by 10-minute incubation in Nuclei Isolation buffer (50 mM Tris [pH 8.0], 60 mM KCl, and 0.5% IGEPAL CA-630) + protease inhibitor cocktail (PIC; Roche) on ice. Pelleted nuclei were dissolved in lysis buffer (0.5% SDS, 10 mM EDTA, 0.5 mM EGTA, 50 mM Tris HCl [pH 8.0]) + PIC and sonicated on a Bioruptor (Diagenode) 40 cycles, max power for 30 seconds followed by 60 seconds of rest. Sonication was followed by pelleting of debris, and then the supernatant was transferred to new tube and chromatin was diluted 5X in dilution buffer (1% Triton, 2 mM EDTA, 150 mM NaCl, 20 mM Tris HCl [pH 8.0] + PIC). Anti-Eomes (ab23345) (10 μg) was used for each ChIP experiment. ChIP was performed over night at 4 °C and subsequently washed and eluted overnight at 65 °C (20 mM Tris HCl [pH 7.5], 5 mM EDTA, 50 mM NaCl, 1% SDS, 100 μg RNase A, and 50 μg proteinase K), treated, and cleaned up using Zymo ChIP DNA Clean & Concentrator before ChIP-seq library preparation. ChIP-seq libraries were prepared by using Followed Rubicon ThruPLEX DNA-seq Kit (CAT. NO. R400427). Single-read sequencing (75-bp) was performed on an Illumina NextSeq500. Reads from high-throughput sequencing were aligned to a mouse reference genome (mm10) using Bowtie. Further analyses were made using the HOMER package (http://homer.ucsd.edu/homer/).

## Supporting information

S1 Fig Submaximal TCR stimulation provides a survival advantage in a model for memory CD8 T-cell formation. (**A**) CD45.1^+^ OT-1 cells (10^4^) were transferred to WT CD45.2^+^ recipients. After 24 hours, mice were infected with LM expressing the indicated peptides. Thirty days after infection, mice were reinfected with LCMV-N4. Shown is the frequency of donor cells in blood, determined by flow cytometry. (**B-I**) OT-1 cells were purified and primed for 30 hours with indicated peptides and anti-CD28. Next, cells were washed and cultured for an additional 5 days with 50 ng/ml IL-15. (**B**) Expression of CD127 and CD25 over time, analyzed by flow cytometry. (**C**) On day 6 after start of stimulation, cells were restimulated with N4 peptide, and their production of IL-2 and IFNγ was assessed after 4 hours by intracellular flow cytometry. (**D**) On day 6 after start of stimulation, cells were labeled with CFSE and restimulated with N4 peptide. After 3 days, proliferation was assessed by flow cytometry. (**E**) To assess their recall capacity in vivo, 5 × 10^4^ in vitro–generated OT-1 memory cells (CD45.2^+^) were transferred into CD45.1/2^+^ recipients. After 25 days, mice were infected with mCMV-N4, and 5 days later, donor-cell expansion in spleen was assessed by flow cytometry. Gated is for CD8 T cells. (**F**) Purified OT-1 cells were primed for 30 hours with 1 ng/ml N4 or Q4 peptides and anti-CD28. Next, cells were washed and cultured for an additional 5 days with 50 ng/ml IL-15 to generate memory cells. RNA was isolated after 0, 30, 72, and 140 hours of culture (*n* = 3). Analysis of *Eomes* and *Tbx21* gene expression by qPCR is shown. (**G,H**) Purified OT-1 cells were stimulated in vitro with anti-CD28 and the indicated concertation of peptides. After 30 hours, relative induction of protein expression of (**G**) CD127, CD122, and CD25 and (**H**) Eomes and T-bet was analyzed by flow cytometry. (**I**) CD45.1^+^ OT-1 cells (5 × 10^5^ in left panel—day 2; or 5 × 10^4^ in right panel—day 4) were transferred in CD45.2^+^ recipients. After 24 hours, mice were infected with mCMV expressing the indicated peptides. Expression in donor cells was analyzed in spleen by flow cytometry. The same data are also shown in Fig 1G, right panel. Shown are representative plots of at least two (**A-F, I**) to four (**G,H,I**) experiments. In (**A,I**), ANOVA followed by Bonferroni posttesting was used; in (**F**), Student *t* test was used to analyze difference between groups. Shown are means ± s.e.m. *P < 0.05, **P < 0.01, ***P < 0.001. Values for each data point can be found in S1 Data. CFSE, carboxyfluorescein succinimidyl ester; IFNγ, interferon gamma; IL, interleukin; LCMV, lymphocytic choriomeningitis virus; LM, *L*. *monocytogenes*; mCMV, murine cytomegalovirus; N4, SIINFEKL; Q4, SIIQFEKL; qPCR, quantitative polymerase chain reaction; TCR, T-cell receptor; WT, wild-type.
(TIF)

S2 Fig Analysis of differential gene expression between maximal and submaximal stimulated OT-1 cells. (**A**) WT (CD45.1^+^) and Eomes^CKO^ (CD45.2^+^) OT-1 cells were mixed in a 1:1 ratio, CFSE labeled, and stimulated in vitro as described under S1B Fig. Proliferation of cells stimulated with the indicated peptides, as determined by CFSE dilution, was followed over time by flow cytometry. Shown is the percentage of cells that has undergone the number of divisions as indicated on the x-axis. Shown are representative plots of at least two experiments using 3 mice per group. (**B**) Eomes^iCKO^ CD8 T cells were stimulated in vitro for 2 days with Poly(I:C). On day 2, cells were sorted for εYFP-positive and εYFP-negative populations (left panel), and expression of Eomes was measured by flow cytometry (right panel). Values for each data point can be found in S1 Data. CFSE, carboxyfluorescein succinimidyl ester; Eomes^CKO^, Eomes^flox/flox^CD4^Cre^; Eomes^iCKO^, Eomes^FL/FL^OT-1^+/-^Rosa^Stop-YFP^Mx1^Cre/+^; WT, wild-type; YFP, yellow fluorescent protein.
(TIF)

S3 Fig Eomes deficiency results in an effector response of increased antigen affinity. (**A**) mBMCs were infected with LCMV and analyzed 7 days later. Percentage of KLRG1^+^ tetramer^+^ cells in spleen, liver, and LN on day 7 after infection was determined by flow cytometry. (**B-I**) mBMCs were infected with mCMV-N4 and analyzed 8 days later. (**B**) Ratio between WT and Eomes^CKO^ K^b^M57^+^ T cells in indicated organs. Dashed line indicates ratio at T_0_. (**C**) IFNγ production after in vitro restimulation of splenocytes with M57 or m139 peptides. (**D**) KLRG1^+^ tetramer^+^ cells in spleen, liver, and LN. (**E**) Quantification of the GeoMean of K^b^M57 and K^b^m139 binding of WT and Eomes^CKO^ tetramer^+^ CD8 T cells in spleen. Representative FACS plot is gated for CD8^+^ donor cells. Arbitrary gates indicate low- and high-tetramer-binding CD8^+^ T cells. (**F**) Splenocytes were stained with K^b^M57 tetramer in the presence of increasing amounts of blocking MHC-I antibodies. The amount of blocking relative to cells stained in absence of antibody is shown. (**G**) Splenocytes were stained with increasing amounts of K^b^m139 tetramer. Percentage of tetramer^+^ cells relative to cells stained with 5 μg/ml is shown. (**H**) Splenocytes were stimulated in vitro with increasing amounts of N4 peptides. Percentage of IFNγ^+^ cells relative to cells stimulated with 100 ng/ml is shown. (**I**) Quantification of the GeoMean of the TCRβ, CD3ε, and CD8α of K^b^M57^+^ and K^b^m139^+^ WT and Eomes^CKO^ CD8^+^ T cells on day 8 after infection of mBMCs with mCMV-N4. (**J**) Quantification of the GeoMean of the TCRβ, CD3ε, and CD8α of D^b^Gp33^+^ WT and Eomes^CKO^ CD8^+^ T cells on day 8 after infection of mBMCs with LCMV. Shown are representative plots of at least two experiments using 3–5 mice per group. Student *t* test was used to analyze differences between groups. Shown are means ± s.e.m. *P < 0.05, **P < 0.01. Values for each data point can be found in S1 Data. FACS, fluorescence-activated cell sorting; GeoMean, geometric mean; IFNγ, interferon gamma; LCMV, lymphocytic choriomeningitis virus; LN, lymph node; M57, SCLEFWQRV; m139, TVYGFCLL; mBMC, mixed bone marrow chimera; mCMV, murine cytomegalovirus; MHC-I, major histocompatibility complex class I; N4, SIINFEKL; TCR, T-cell receptor; WT, wild-type.
(TIF)

S4 Fig Eomes promotes survival of low-affinity cells into the memory phase. (**A**) Mixed bone marrow chimeras were generated using WT (CD45.1^+^) and Eomes^Flox/Flox^ (CD45.2^+^) cells in WT B6 recipients (CD45.1/2^+^). After reconstitution, mice were infected with LCMV. (Left) The ratio between D^b^Gp33^+^ cells was followed over time in the blood by flow cytometry. (Middle) The GeoMean of D^b^Gp33 staining within antigen-specific donor populations was determined by flow cytometry. (Right) After 38 days, splenocytes were stained with increasing amounts of K^b^m139 tetramer. The percentage of D^b^Gp33^+^ cells relative to cells stained with 5 μg/ml is shown. (**B-G**) Mixed bone marrow chimeras were generated using WT (CD45.1^+^) and Eomes^CKO^ (CD45.2^+^) cells in WT B6 recipients (CD45.1/2^+^). (**B**) Mice were infected with LCMV. The percentage of D^b^Gp33^Bright^ cells was determined within the total pool of D^b^Gp33^+^ cells by arbitrary gating. Stars show significant differences between groups per time point. *P* values show significance of linear regression within the indicated group. (**C-G**) Mice were infected with mCMV-N4. (**C**) Analysis of the ratio between WT and Eomes^CKO^ K^b^M57^+^ cells in the blood. (**D-F**) Quantification of the GeoMean of (**D**) K^b^m57 staining and (**E**) T-bet staining of WT and Eomes^CKO^ tetramer^+^ CD8 T cells in blood. Dashed line indicates the ratio between total donor CD8 T cells before infection. (**F**) GeoMean of CD5 staining on K^b^m139^+^ cells in spleen on day 45 after infection. (**G,H**) On day 58 after infection, (**F**) the GeoMean of K^b^m139 staining of WT and Eomes^CKO^ tetramer^+^ CD8 T cells was determined in spleen. FACS plot shows representative plot gated for donor CD8^+^ cells. (**G**) Five weeks after infection, CD8 T cells were purified from spleens, and 3 × 10^6^ cells were transferred to CD45.2^+^ recipients. After 24 hours, mice were infected with mCMV and intensity of tetramer staining of CD8 T cells was determined by flow cytometry after 6 days in spleen. Shown are representative plots of at least two experiments using 4–6 mice per group. Student *t* test was used to analyze differences between groups. For (**B**), linear regression was performed to correlate affinity and time. Shown are means ± s.e.m. *P < 0.05, **P < 0.01, ***P < 0.001. Values for each data point can be found in S1 Data. B6, C57BL/6; Eomes^CKO^, Eomes^flox/flox^ CD4Cre; FACS, fluorescence-activated cell sorting; GeoMean, geometric mean; LCMV, lymphocytic choriomeningitis virus; M57, SCLEFWQRV; mCMV, murine cytomegalovirus; N4, SIINFEKL; WT, wild-type.
(TIF)

S5 Fig Memory precursors of suboptimal affinity express higher Bcl-2 protein levels. (**A,B**) OT-1 cells were purified and primed for 30 hours with 1 ng/ml of the indicated peptides and anti-CD28. Next, cells were washed and cultured for an additional 5 days with 50 ng/ml IL-15. Shown is quantification of the geometric mean of (**A**) CFSE signal and (**B**) Ki67 staining over time. (**C**) OT-1 cells were purified and primed for 30 hours with the indicated concentrations of peptides and anti-CD28. On day 6 after start of stimulation, apoptosis was assessed by flow cytometry, using annexin V and live/dead dye. x-Axis shows the concentration of peptide used for priming in the first 30 hours. (**D**) Microarray data of the experiment described in Fig 1D and 1E were analyzed for differential expression of 619 genes associated with apoptosis at 140 hours of culture. Shown are the top-50 differentially expressed genes. (**E**) CD45.1^+^ OT-1 cells were transferred in WT (CD45.2^+^) recipients. After 24 hours, mice were infected with mCMV expressing the indicated peptides. The geometric mean of Bcl-2 in donor cells was determined on day 4 after infection in spleen. The same data are also shown in Fig 4H. (**F**) Memory T cells were generated in vitro using 1 ng/ml of N4 or APLs as described for S1B Fig. On day 6 of stimulation, 1 × 10^6^ OT-1 cells (CD45.1^+^) were transferred to WT recipients (CD45.2^+^), and on indicated time points after transfer, splenocytes were isolated and expression of Eomes and Bcl-2 was quantified by flow cytometry. Shown is the correlation between expression of Eomes and Bcl-2. (**G**) Memory T cells were generated separately in vitro using 1 ng/ml of N4 peptide for priming of CD45.1^+^ or A2/Q4 peptide for priming of CD45.1/2^+^ OT-1 cells as described for S1B Fig. On day 6 of stimulation, cells were mixed in equal numbers (N4-primed with A2- or Q4-primed), and 1 × 10^6^ cells were subsequently transferred to WT recipients (CD45.2^+^). Twenty-four hours after transfer, the ratio between donor cells in spleen and liver was determined by flow cytometry. (**H**) Memory OT-1 cells were generated in vitro after priming with N4, A2, or Q4 peptides using CD45.1^+^ or CD45.1/2^+^ cells, respectively. On day 6 of stimulation, cells were mixed in equal numbers (N4-primed with A2- or Q4-primed) and labeled with CFSE, and 1.4 × 10^6^ cells were subsequently transferred to WT recipients (CD45.2^+^). Proliferation of donor cells was assessed by analysis of CFSE dilution via flow cytometry. Shown are representative plots of at least two (**A-C,E-H**) experiments. (**D**) shows data from microarray analysis of three biologically independent samples, each pooled from 3–4 mice. In (**A-C,E**), ANOVA followed by Bonferroni posttesting and, in (**G,H**), Student *t* test were used to analyze differences between groups. In (**F**), linear regression was used to determine significance of correlations. Shown are means ± s.e.m. *P < 0.05, **P < 0.01, ***P < 0.001. Values for each data point can be found in S1 Data. A2, SAINFEKL; APL, altered peptide ligand; CFSE, carboxyfluorescein succinimidyl ester; IL, interleukin; mCMV, murine cytomegalovirus; N4, SIINFEKL; Q4, SIIQFEKL; WT, wild-type.
(TIF)

S6 Fig Eomes directly drives expression of Bcl-2. (**A-C**) OT-1 cells were stimulated for 30 hours with N4 peptide, and DNA binding of Eomes was determined by ChIP-seq. (**A**) Binding motifs enriched in the ChIP-seq analysis. (**B**) Binding of Eomes to the loci of *Prf1* and *Ncr1*. (**C**) qPCR analysis of *Bcl2* promoter regions as indicated in Fig 5A using activated OT-1 cells with low or high amounts of Eomes protein content. Eomes content was determined by flow cytometry. (**D**) OT-1 (CD45.1/2^+^) and Eomes^CKO^ OT-1 (CD45.2^+^) cells were mixed in a 1:1 ratio, and 10,000 cells were transferred in WT (CD45.1^+^) recipients. Mice were infected with LM-N4, LM-Q4, or LM-V4. Expression of Bcl-2 was determined in donor-cell MPECs (CD127^+^KLRG1^-^) in the blood at day 35 after infection. (**A-C**) shows data from two biologically independent samples, each pooled from 3 mice. (**D**) shows a representative plot of two independent experiments. In (**D**), ANOVA followed by Bonferroni posttesting was used to analyze difference between groups. Shown are means ± s.e.m. *P < 0.05. Values for each data point can be found in S1 Data. ChIP-seq, chromatin immunoprecipitation sequencing; LM, *L*. *monocytogenes*; MPEC, memory precursor effector cell; N4, SIINFEKL; qPCR, quantitative polymerase chain reaction; Q4, SIIQFEKL; V4, SIIVFEKL; WT, wild-type.
(TIF)

S7 Fig Low-affinity CD8 T cells depend on Bcl-2 for their survival. (**A**) Purified OT-1 cells were cultured with the indicated peptides and anti-CD28 in the presence of increasing amounts of ABT-199. After 30 hours, viability was analyzed by flow cytometry. (**B,C**) WT mice were infected with mCMV. After (**B**) 3 or (**C**) 6 days, animals received the indicated dose of ABT-199. On day 7, splenocytes were stained with increasing amounts of K^b^m139 tetramer. Percentage of tetramer^+^ cells in the MPECs (CD127^+^KLRG1^-^) relative to cells stained with 5 μg/ml is shown. Representative FACS plots are gated for CD8^+^ cells. (**D-F**) mBMCs were generated using WT (CD45.1^+^) and Eomes^CKO^ (CD45.2^+^) cells in WT (CD45.1/2^+^) recipients. (**D,E**) Eight weeks after reconstitution, animals were infected with mCMV-N4. Six days after infection, mice received a single injection i.p. with ABT-199 (+ABT) or carrier only (-ABT). (**D**) After 7 days, the GeoMeans of K^b^m139^+^ and K^b^M57^+^ staining in spleen were analyzed by flow cytometry. Representative FACS plots show K^b^M57^+^ staining. Gated is for donor K^b^M57^+^ cells. (**E**) After 41 days, splenocytes were stained with increasing amounts of K^b^m139 tetramer, and the frequency of positive cells was analyzed by flow cytometry. Shown is the fraction of K^b^m139^+^ cells relative to staining with maximum concentration. (**F**) mBMCs were infected with LCMV. Thirty days after infection, WT and Eomes^CKO^ CD8^+^D^b^Gp33^+^ cells were sorted and analyzed by TCR sequencing. Shown is the number of highly expanded clones (>5% of the total population) within the Vβ13.2^+^ family. (**G,H**) Naïve EYFP^+^Eomes^CKO^ OT-1 (CD45.2) cells were transduced with a lentiviral construct to overexpress Bcl-2 and RFP. RFP^+^EYFP^+^Eomes^CKO^ OT-1 cells were sorted, mixed in a 1:1:1 ratio with EYFP^+^Eomes^CKO^ OT-1 (CD45.2) and OT-1 (CD45.1/2) cells and transferred to WT (CD45.1) recipients. After 24 hours, animals were infected with LM-Q4 and, 30 days later, were reinfected with mCMV-N4. Seven days after secondary infection (**G**), Bcl-2 levels were quantified in donor-cell populations. (**H**) Donor cells were restimulated in vitro with N4 peptides, and IFNγ production was quantified by intracellular flow cytometry. Shown are data from two (**B-H**) or three (**A**) experiments. In (**A-C,E,G,H**), ANOVA followed by Bonferroni posttesting and, in (**D,F**), Student *t* test were used to analyze differences between groups. Shown are means ± s.e.m. *P < 0.05, **P < 0.01, ***P < 0.001. Values for each data point can be found in S1 Data. FACS, fluorescence-activated cell sorting; GeoMean, geometric mean; IFNγ, interferon gamma; LCMV, lymphocytic choriomeningitis virus; LM, *L*. *monocytogenes*; M57, SCLEFWQRV; mBMC, mixed bone marrow chimera; mCMV, murine cytomegalovirus; MPEC, memory precursor effector cell; N4, SIINFEKL; Q4, SIIQFEKL; RFP, red fluorescent protein; TCR, T-cell receptor; WT, wild-type.
(TIF)

S8 Fig Eomes promotes the cross-reactive potential of the memory CD8 T-cell pool. (**A**) mBMCs were infected with PR8, and antigen-specific cells were analyzed after 10 days. The GeoMean of K^b^METM binding of WT and Eomes^CKO^ tetramer^+^ CD8 T cells in spleen is quantified. (**B,C**) mBMCs were infected with influenza HK2/68. At the indicated time points, lymphocytes in blood were restimulated with METM or MDAM peptides, and after 4 hours, IFNγ was measured. Shown is (**B**) the ratio between IFNγ^+^ cells after METM or MDAM stimulation and (**C**) the fraction of CD127^+^ cells within the IFNγ^+^ donor-cell populations after METM (low-affinity) and MDAM (high-affinity) stimulation for WT and Eomes^CKO^ cells. Shown are representative plots of at least two experiments using 4–6 mice per group. Student *t* test was used to analyze differences between groups. Shown are means ± s.e.m. *P < 0.05, **P < 0.01, ***P < 0.001. Values for each data point can be found in S1 Data. Eomes^CKO^, Eomes^flox/flox^ CD4Cre; GeoMean, geometric mean; IFNγ, interferon gamma; mBMC, mixed bone marrow chimera; MDAM, ASNENMDAM; METM, ASNENMETM; PR8, influenza A strain PR/8/34; WT, wild-type.
(TIF)

S1 Table Affinity-dependent differential gene expression in activated CD8 T cells. Purified OT-1 cells were primed for 30 hours with 1 ng/ml N4 or Q4 peptides and anti-CD28. Next, cells were washed and cultured for an additional 5 days with 50 ng/ml IL-15 to generate memory cells. RNA was isolated after 0, 30, 72, and 140 hours of culture (*n* = 3). Displayed is a list of differentially expressed genes per time point. IL, interleukin; N4, SIINFEKL; Q4, SIIQFEKL.
(XLSX)

S1 Raw Images Raw data images of blots shown in main Fig 4F. Samples for WB were lysed in Laemmli lysis buffer (0.12 M Tris HCL [pH 8], 4% SDS, 20% glycerol, 0.05 μg/μL bromophenol blue, and 50 mM dithiothreitol) and boiled for 5 min. Protein contents were determined by the Bio-Rad protein assay (Bio-Rad Laboratories), and equal amounts of total lysate were analyzed by 12% SDS–polyacrylamide gel electrophoresis. Each sample was loaded on three separate blots. Proteins were transferred to Immobilon-P and incubated with blocking buffer (TBS-T) containing 2% low-fat milk for 1 hour. Blots were then cut according to protein markers to allow analysis of multiple proteins from a single membrane. Next, membranes were incubated with an antibody against Mcl-1 (BD Pharmingen), Bcl-XL (Transduction Laboratories), Bim (Stressgen Bioreagents), Bcl-2 (Enzo Lifesciences), p100/p52 (Cell Signaling), or β-actin (Santa Cruz Biotechnology) overnight at 4 °C in TBS-T. Blots labeled with β-actin were incubated with IRDye 800. All other antibodies were labeled with IRDye 680 (Both Li-Cor) for 1 hour. Odyssey Imager (Li-Cor) was used as a detection method. IRDye 680 was visualized at 700 nm and IRDye 800 at 800 nm. “X” indicates irrelevant sample or irrelevant membrane. TBS-T, Tris buffered saline/Tween-20; WB, western blot.
(PDF)

S1 Data Excel spreadsheet containing, in separate sheets, the underlying numerical data for all figure panels. (XLSX)

## Abbreviations

A2: SAINFEKL
APL: altered peptide ligand
B6: C57BL/6
CFSE: carboxyfluorescein succinimidyl ester
ChIP: chromatin immunoprecipitation
ChIP-seq: ChIP sequencing
DL: detection limit
Eomes^CKO^: Eomes^flox/flox^ CD4Cre
Eomes^iCKO^: Eomes^flox/flox^Rosa^Stop-EYFP^Mx1^Cre^OT-1
eYFP: enhanced yellow fluorescent protein
IL: interleukin
LCMV: lymphocytic choriomeningitis virus
LM: *Listeria monocytogenes*
mBMC: mixed bone marrow chimera
mCMV: murine cytomegalovirus
MDAM: ASNENMDAM
METM: ASNENMETM
MPEC: memory precursor effector cell
n.s.: not significant
N4: SIINFEKL
Poly(I:C): polyinosine-polycytidylic acid
PR8: influenza A strain PR/8/34
Q4: SIIQFEKL
qPCR: quantitative polymerase chain reaction
RU: relative units
T4: SIITFEKL
TCR: T-cell receptor
T_RM_: tissue-resident memory T cell
V4: SIIVFEKL
VM: virtual memory cell
WT: wild-type
Y3: SIYNFEKL
