# Supplementary figures and images for: Eomes broadens the scope of CD8 T-cell memory by inhibiting apoptosis in cells of low affinity
Source: PLoS Biol. 2020 Mar 17;18(3):e3000648. doi: 10.1371/journal.pbio.3000648 (PMC7077837; doi:10.1371/journal.pbio.3000648)

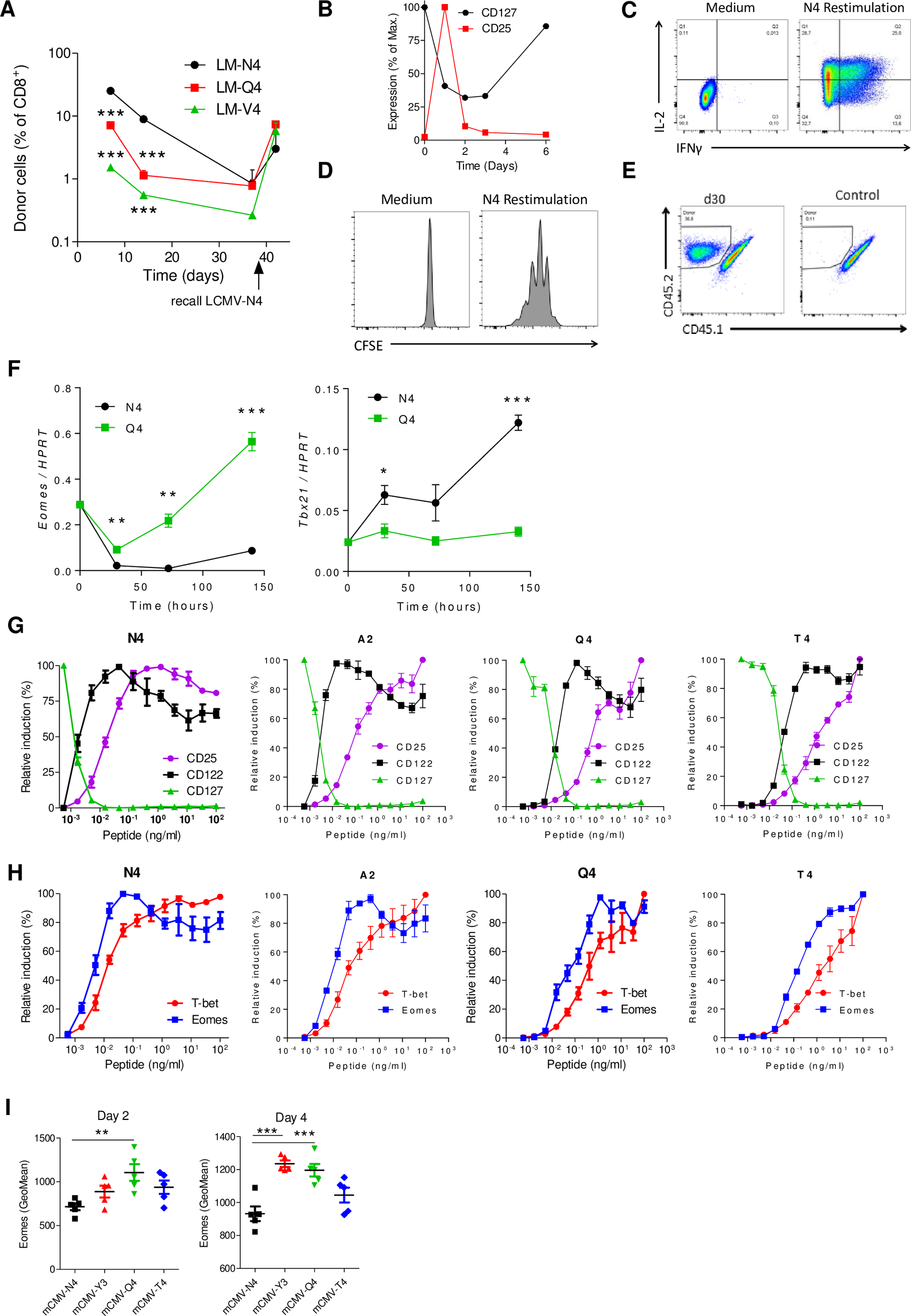

Supplement: S1 Fig — (A) CD45.1+ OT-1 cells (104) were transferred to WT CD45.2+ recipients. After 24 hours, mice were infected with LM expressing the indicated peptides. Thirty days after infection, mice were reinfected with LCMV-N4. Shown is the frequency of donor cells in blood, determined by flow cytometry. (B-I) OT-1 cells were purified and primed for 30 hours with indicated peptides and anti-CD28. Next, cells were washed and cultured for an additional 5 days with 50 ng/ml IL-15. (B) Expression of CD127 and CD25 over time, analyzed by flow cytometry. (C) On day 6 after start of stimulation, cells were restimulated with N4 peptide, and their production of IL-2 and IFNγ was assessed after 4 hours by intracellular flow cytometry. (D) On day 6 after start of stimulation, cells were labeled with CFSE and restimulated with N4 peptide. After 3 days, proliferation was assessed by flow cytometry. (E) To assess their recall capacity in vivo, 5 × 10^4^ in vitro–generated OT-1 memory cells (CD45.2+) were transferred into CD45.1/2+ recipients. After 25 days, mice were infected with mCMV-N4, and 5 days later, donor-cell expansion in spleen was assessed by flow cytometry. Gated is for CD8 T cells. (F) Purified OT-1 cells were primed for 30 hours with 1 ng/ml N4 or Q4 peptides and anti-CD28. Next, cells were washed and cultured for an additional 5 days with 50 ng/ml IL-15 to generate memory cells. RNA was isolated after 0, 30, 72, and 140 hours of culture (n = 3). Analysis of Eomes and Tbx21 gene expression by qPCR is shown. (G,H) Purified OT-1 cells were stimulated in vitro with anti-CD28 and the indicated concertation of peptides. After 30 hours, relative induction of protein expression of (G) CD127, CD122, and CD25 and (H) Eomes and T-bet was analyzed by flow cytometry. (I) CD45.1+ OT-1 cells (5 × 10^5^ in left panel—day 2; or 5 × 10^4^ in right panel—day 4) were transferred in CD45.2+ recipients. After 24 hours, mice were infected with mCMV expressing the indicated peptides. Expression in donor cell [file pbio.3000648.s001.tif]

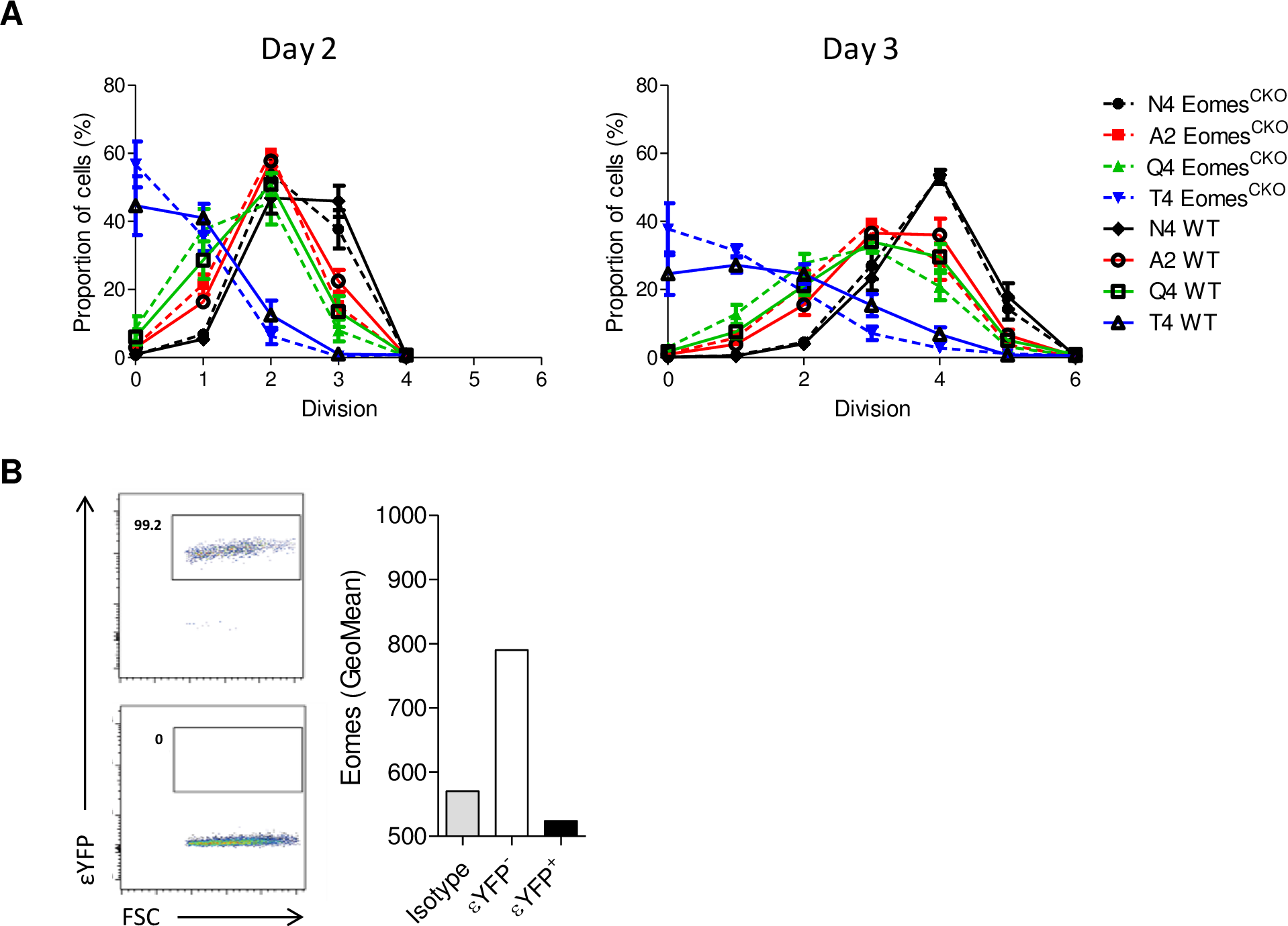

Supplement: S2 Fig — (A) WT (CD45.1+) and EomesCKO (CD45.2+) OT-1 cells were mixed in a 1:1 ratio, CFSE labeled, and stimulated in vitro as described under S1B Fig. Proliferation of cells stimulated with the indicated peptides, as determined by CFSE dilution, was followed over time by flow cytometry. Shown is the percentage of cells that has undergone the number of divisions as indicated on the x-axis. Shown are representative plots of at least two experiments using 3 mice per group. (B) EomesiCKO CD8 T cells were stimulated in vitro for 2 days with Poly(I:C). On day 2, cells were sorted for εYFP-positive and εYFP-negative populations (left panel), and expression of Eomes was measured by flow cytometry (right panel). Values for each data point can be found in S1 Data. CFSE, carboxyfluorescein succinimidyl ester; EomesCKO, Eomesflox/floxCD4Cre; EomesiCKO, EomesFL/FLOT-1+/-RosaStop-YFPMx1Cre/+; WT, wild-type; YFP, yellow fluorescent protein. (TIF) [file pbio.3000648.s002.tif]

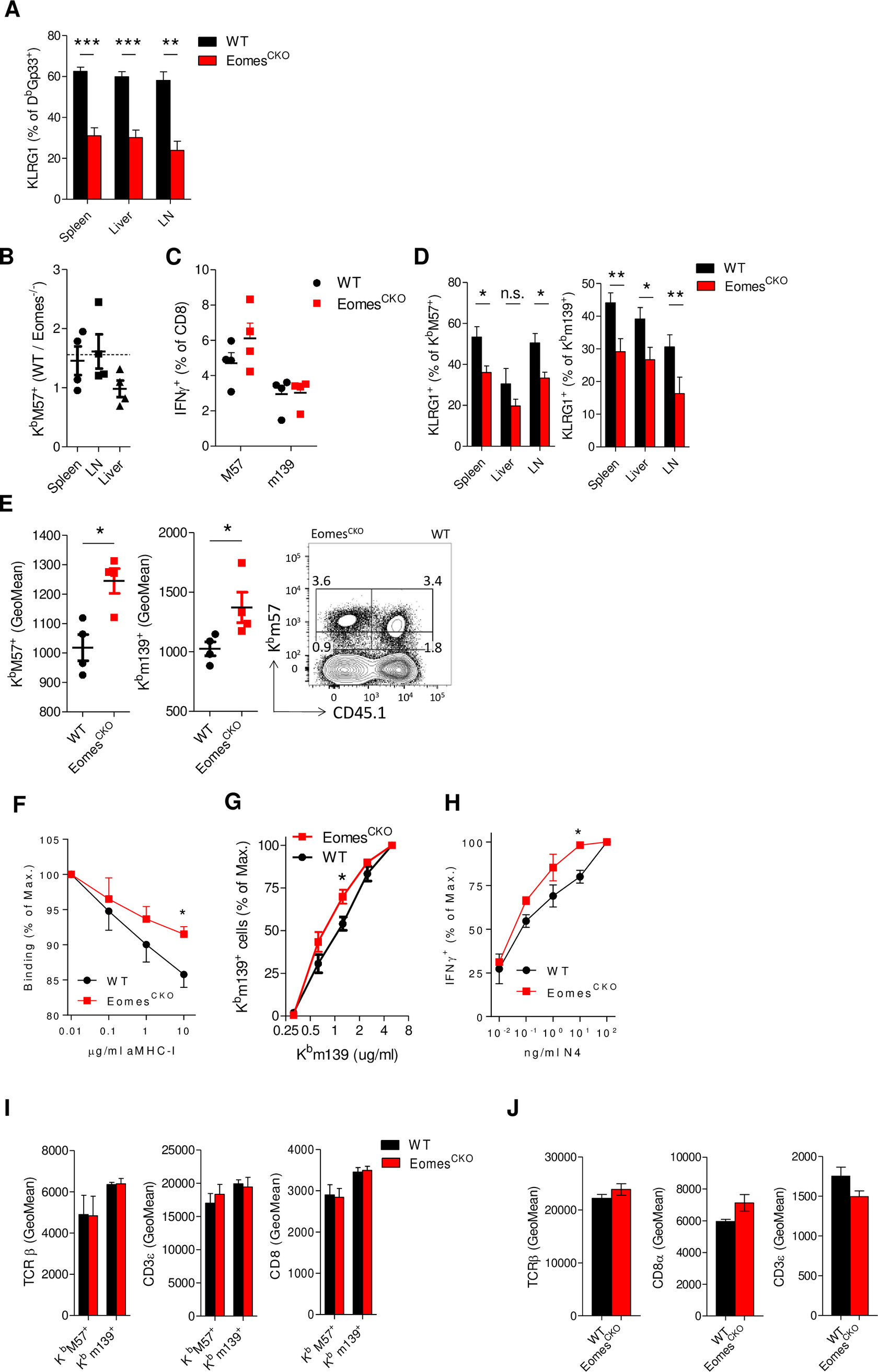

Supplement: S3 Fig — (A) mBMCs were infected with LCMV and analyzed 7 days later. Percentage of KLRG1+ tetramer+ cells in spleen, liver, and LN on day 7 after infection was determined by flow cytometry. (B-I) mBMCs were infected with mCMV-N4 and analyzed 8 days later. (B) Ratio between WT and EomesCKO KbM57+ T cells in indicated organs. Dashed line indicates ratio at T0. (C) IFNγ production after in vitro restimulation of splenocytes with M57 or m139 peptides. (D) KLRG1+ tetramer+ cells in spleen, liver, and LN. (E) Quantification of the GeoMean of KbM57 and Kbm139 binding of WT and EomesCKO tetramer+ CD8 T cells in spleen. Representative FACS plot is gated for CD8+ donor cells. Arbitrary gates indicate low- and high-tetramer-binding CD8+ T cells. (F) Splenocytes were stained with KbM57 tetramer in the presence of increasing amounts of blocking MHC-I antibodies. The amount of blocking relative to cells stained in absence of antibody is shown. (G) Splenocytes were stained with increasing amounts of Kbm139 tetramer. Percentage of tetramer+ cells relative to cells stained with 5 μg/ml is shown. (H) Splenocytes were stimulated in vitro with increasing amounts of N4 peptides. Percentage of IFNγ+ cells relative to cells stimulated with 100 ng/ml is shown. (I) Quantification of the GeoMean of the TCRβ, CD3ε, and CD8α of KbM57+ and Kbm139+ WT and EomesCKO CD8+ T cells on day 8 after infection of mBMCs with mCMV-N4. (J) Quantification of the GeoMean of the TCRβ, CD3ε, and CD8α of DbGp33+ WT and EomesCKO CD8+ T cells on day 8 after infection of mBMCs with LCMV. Shown are representative plots of at least two experiments using 3–5 mice per group. Student t test was used to analyze differences between groups. Shown are means ± s.e.m. *P < 0.05, **P < 0.01. Values for each data point can be found in S1 Data. FACS, fluorescence-activated cell sorting; GeoMean, geometric mean; IFNγ, interferon gamma; LCMV, lymphocytic choriomeningitis virus; LN, lymph node; M57, SCLEFWQRV; m139, TVYGFCLL; mBMC, mixed b [file pbio.3000648.s003.tif]

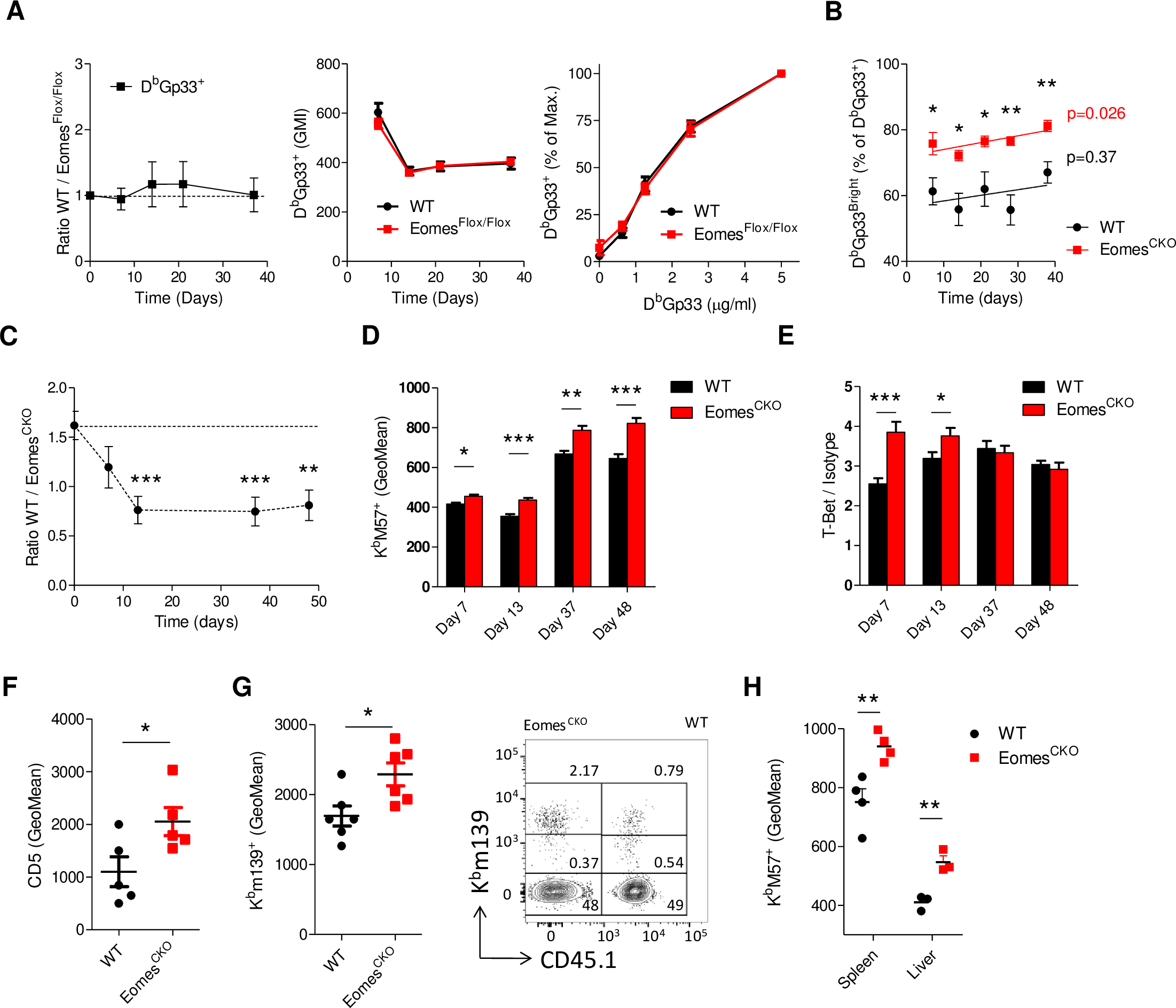

Supplement: S4 Fig — (A) Mixed bone marrow chimeras were generated using WT (CD45.1+) and EomesFlox/Flox (CD45.2+) cells in WT B6 recipients (CD45.1/2+). After reconstitution, mice were infected with LCMV. (Left) The ratio between DbGp33+ cells was followed over time in the blood by flow cytometry. (Middle) The GeoMean of DbGp33 staining within antigen-specific donor populations was determined by flow cytometry. (Right) After 38 days, splenocytes were stained with increasing amounts of Kbm139 tetramer. The percentage of DbGp33+ cells relative to cells stained with 5 μg/ml is shown. (B-G) Mixed bone marrow chimeras were generated using WT (CD45.1+) and EomesCKO (CD45.2+) cells in WT B6 recipients (CD45.1/2+). (B) Mice were infected with LCMV. The percentage of DbGp33Bright cells was determined within the total pool of DbGp33+ cells by arbitrary gating. Stars show significant differences between groups per time point. P values show significance of linear regression within the indicated group. (C-G) Mice were infected with mCMV-N4. (C) Analysis of the ratio between WT and EomesCKO KbM57+ cells in the blood. (D-F) Quantification of the GeoMean of (D) Kbm57 staining and (E) T-bet staining of WT and EomesCKO tetramer+ CD8 T cells in blood. Dashed line indicates the ratio between total donor CD8 T cells before infection. (F) GeoMean of CD5 staining on Kbm139+ cells in spleen on day 45 after infection. (G,H) On day 58 after infection, (F) the GeoMean of Kbm139 staining of WT and EomesCKO tetramer+ CD8 T cells was determined in spleen. FACS plot shows representative plot gated for donor CD8+ cells. (G) Five weeks after infection, CD8 T cells were purified from spleens, and 3 × 10^6^ cells were transferred to CD45.2+ recipients. After 24 hours, mice were infected with mCMV and intensity of tetramer staining of CD8 T cells was determined by flow cytometry after 6 days in spleen. Shown are representative plots of at least two experiments using 4–6 mice per group. Student t test was used to analyze di [file pbio.3000648.s004.tif]

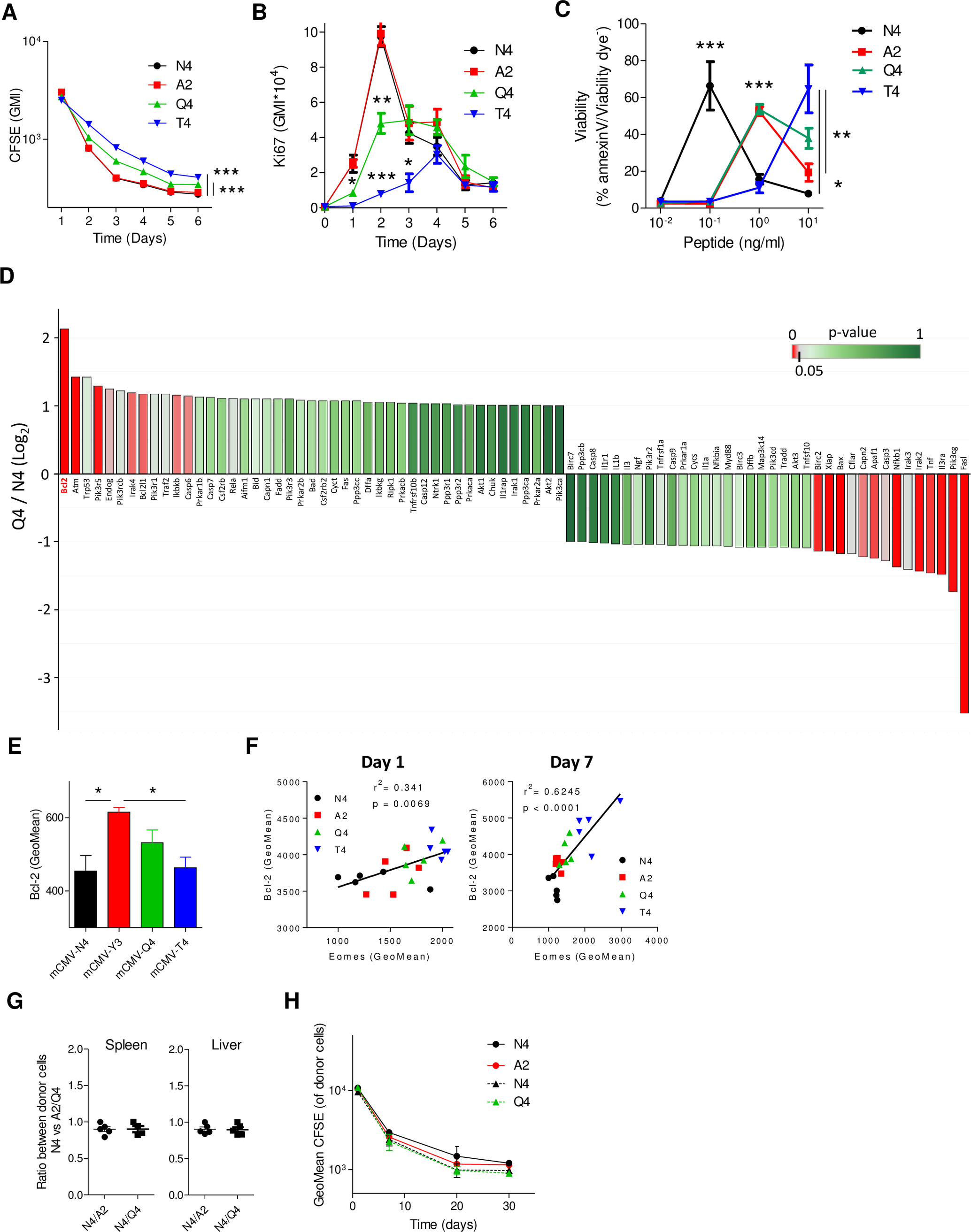

Supplement: S5 Fig — (A,B) OT-1 cells were purified and primed for 30 hours with 1 ng/ml of the indicated peptides and anti-CD28. Next, cells were washed and cultured for an additional 5 days with 50 ng/ml IL-15. Shown is quantification of the geometric mean of (A) CFSE signal and (B) Ki67 staining over time. (C) OT-1 cells were purified and primed for 30 hours with the indicated concentrations of peptides and anti-CD28. On day 6 after start of stimulation, apoptosis was assessed by flow cytometry, using annexin V and live/dead dye. x-Axis shows the concentration of peptide used for priming in the first 30 hours. (D) Microarray data of the experiment described in Fig 1D and 1E were analyzed for differential expression of 619 genes associated with apoptosis at 140 hours of culture. Shown are the top-50 differentially expressed genes. (E) CD45.1+ OT-1 cells were transferred in WT (CD45.2+) recipients. After 24 hours, mice were infected with mCMV expressing the indicated peptides. The geometric mean of Bcl-2 in donor cells was determined on day 4 after infection in spleen. The same data are also shown in Fig 4H. (F) Memory T cells were generated in vitro using 1 ng/ml of N4 or APLs as described for S1B Fig. On day 6 of stimulation, 1 × 10^6^ OT-1 cells (CD45.1+) were transferred to WT recipients (CD45.2+), and on indicated time points after transfer, splenocytes were isolated and expression of Eomes and Bcl-2 was quantified by flow cytometry. Shown is the correlation between expression of Eomes and Bcl-2. (G) Memory T cells were generated separately in vitro using 1 ng/ml of N4 peptide for priming of CD45.1+ or A2/Q4 peptide for priming of CD45.1/2+ OT-1 cells as described for S1B Fig. On day 6 of stimulation, cells were mixed in equal numbers (N4-primed with A2- or Q4-primed), and 1 × 10^6^ cells were subsequently transferred to WT recipients (CD45.2+). Twenty-four hours after transfer, the ratio between donor cells in spleen and liver was determined by flow cytometry. (H) Memory OT-1 cells w [file pbio.3000648.s005.tif]

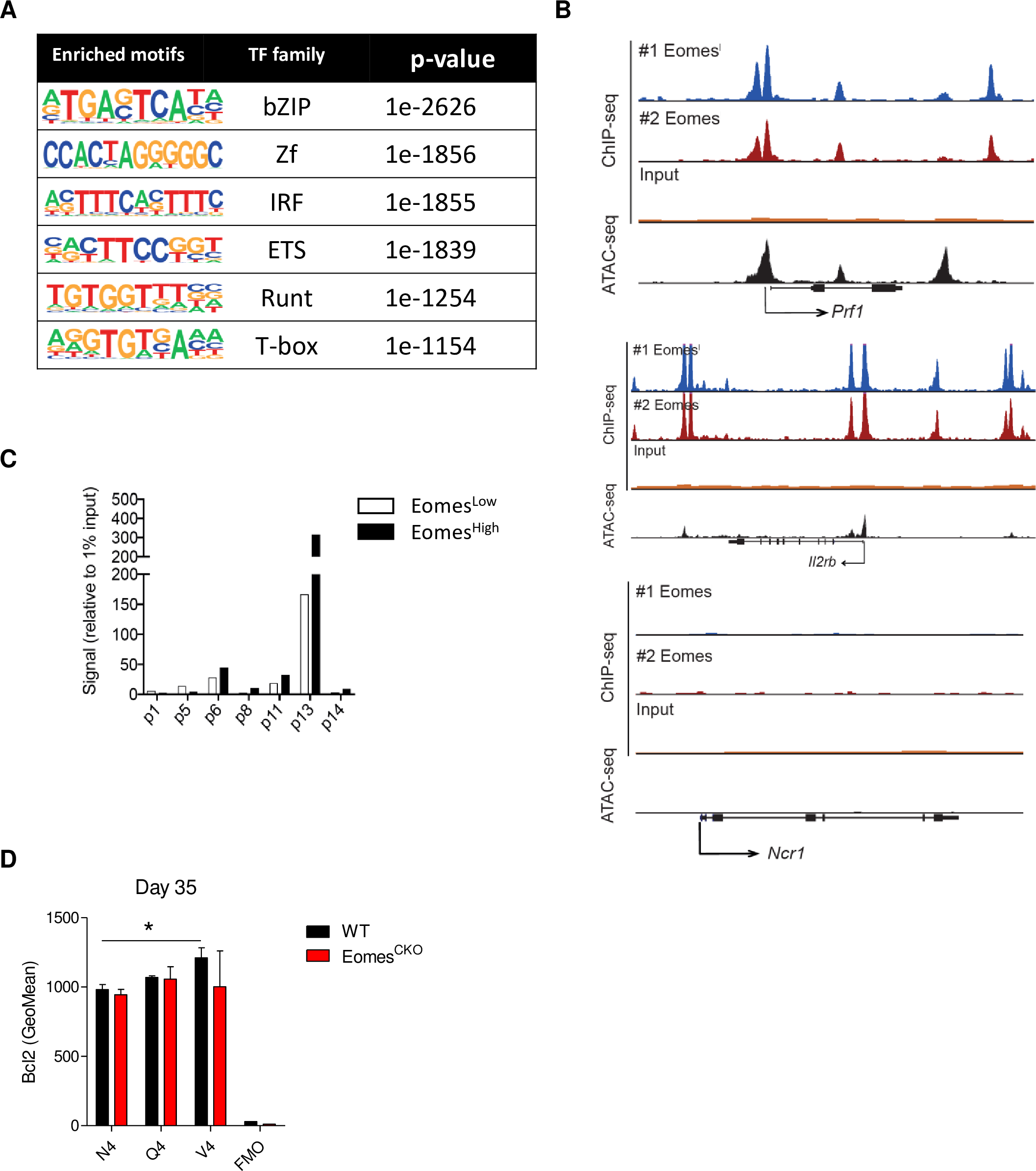

Supplement: S6 Fig — (A-C) OT-1 cells were stimulated for 30 hours with N4 peptide, and DNA binding of Eomes was determined by ChIP-seq. (A) Binding motifs enriched in the ChIP-seq analysis. (B) Binding of Eomes to the loci of Prf1 and Ncr1. (C) qPCR analysis of Bcl2 promoter regions as indicated in Fig 5A using activated OT-1 cells with low or high amounts of Eomes protein content. Eomes content was determined by flow cytometry. (D) OT-1 (CD45.1/2+) and EomesCKO OT-1 (CD45.2+) cells were mixed in a 1:1 ratio, and 10,000 cells were transferred in WT (CD45.1+) recipients. Mice were infected with LM-N4, LM-Q4, or LM-V4. Expression of Bcl-2 was determined in donor-cell MPECs (CD127+KLRG1-) in the blood at day 35 after infection. (A-C) shows data from two biologically independent samples, each pooled from 3 mice. (D) shows a representative plot of two independent experiments. In (D), ANOVA followed by Bonferroni posttesting was used to analyze difference between groups. Shown are means ± s.e.m. *P < 0.05. Values for each data point can be found in S1 Data. ChIP-seq, chromatin immunoprecipitation sequencing; LM, L. monocytogenes; MPEC, memory precursor effector cell; N4, SIINFEKL; qPCR, quantitative polymerase chain reaction; Q4, SIIQFEKL; V4, SIIVFEKL; WT, wild-type. (TIF) [file pbio.3000648.s006.tif]

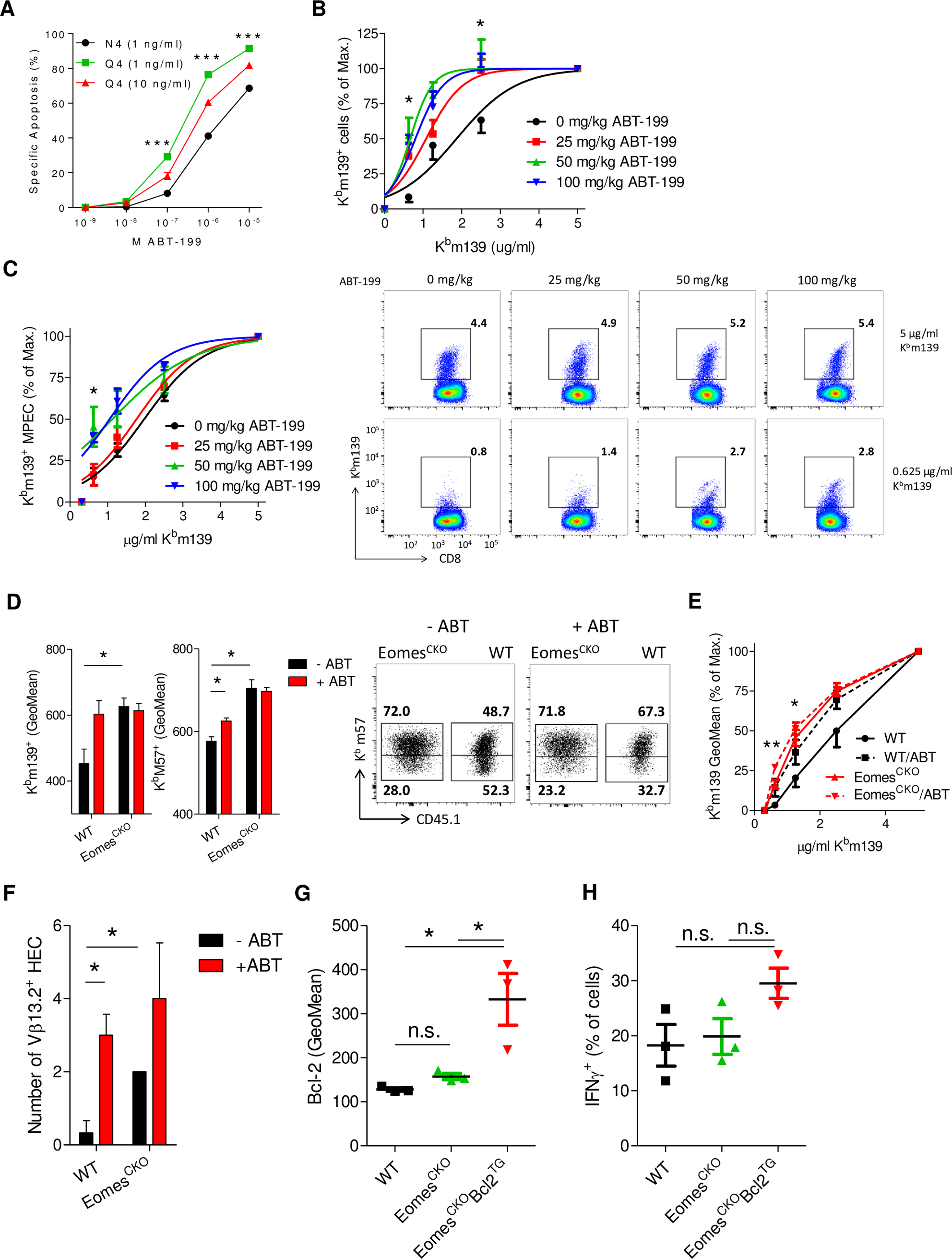

Supplement: S7 Fig — (A) Purified OT-1 cells were cultured with the indicated peptides and anti-CD28 in the presence of increasing amounts of ABT-199. After 30 hours, viability was analyzed by flow cytometry. (B,C) WT mice were infected with mCMV. After (B) 3 or (C) 6 days, animals received the indicated dose of ABT-199. On day 7, splenocytes were stained with increasing amounts of Kbm139 tetramer. Percentage of tetramer+ cells in the MPECs (CD127+KLRG1-) relative to cells stained with 5 μg/ml is shown. Representative FACS plots are gated for CD8+ cells. (D-F) mBMCs were generated using WT (CD45.1+) and EomesCKO (CD45.2+) cells in WT (CD45.1/2+) recipients. (D,E) Eight weeks after reconstitution, animals were infected with mCMV-N4. Six days after infection, mice received a single injection i.p. with ABT-199 (+ABT) or carrier only (-ABT). (D) After 7 days, the GeoMeans of Kbm139+ and KbM57+ staining in spleen were analyzed by flow cytometry. Representative FACS plots show KbM57+ staining. Gated is for donor KbM57+ cells. (E) After 41 days, splenocytes were stained with increasing amounts of Kbm139 tetramer, and the frequency of positive cells was analyzed by flow cytometry. Shown is the fraction of Kbm139+ cells relative to staining with maximum concentration. (F) mBMCs were infected with LCMV. Thirty days after infection, WT and EomesCKO CD8+DbGp33+ cells were sorted and analyzed by TCR sequencing. Shown is the number of highly expanded clones (>5% of the total population) within the Vβ13.2+ family. (G,H) Naïve EYFP+EomesCKO OT-1 (CD45.2) cells were transduced with a lentiviral construct to overexpress Bcl-2 and RFP. RFP+EYFP+EomesCKO OT-1 cells were sorted, mixed in a 1:1:1 ratio with EYFP+EomesCKO OT-1 (CD45.2) and OT-1 (CD45.1/2) cells and transferred to WT (CD45.1) recipients. After 24 hours, animals were infected with LM-Q4 and, 30 days later, were reinfected with mCMV-N4. Seven days after secondary infection (G), Bcl-2 levels were quantified in donor-cell populations. (H) Donor ce [file pbio.3000648.s007.tif]

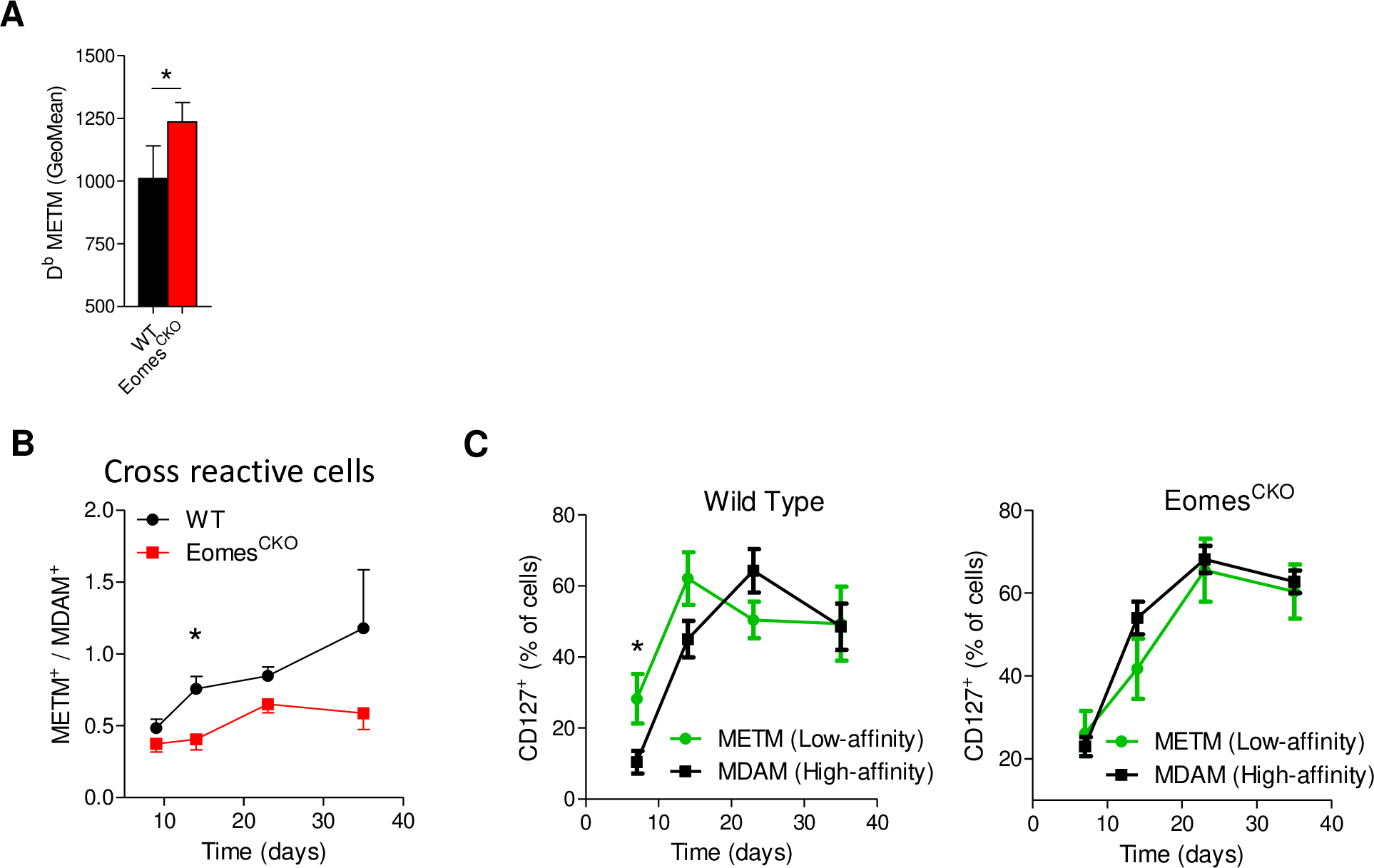

Supplement: S8 Fig — (A) mBMCs were infected with PR8, and antigen-specific cells were analyzed after 10 days. The GeoMean of KbMETM binding of WT and EomesCKO tetramer+ CD8 T cells in spleen is quantified. (B,C) mBMCs were infected with influenza HK2/68. At the indicated time points, lymphocytes in blood were restimulated with METM or MDAM peptides, and after 4 hours, IFNγ was measured. Shown is (B) the ratio between IFNγ+ cells after METM or MDAM stimulation and (C) the fraction of CD127+ cells within the IFNγ+ donor-cell populations after METM (low-affinity) and MDAM (high-affinity) stimulation for WT and EomesCKO cells. Shown are representative plots of at least two experiments using 4–6 mice per group. Student t test was used to analyze differences between groups. Shown are means ± s.e.m. *P < 0.05, **P < 0.01, ***P < 0.001. Values for each data point can be found in S1 Data. EomesCKO, Eomesflox/flox CD4Cre; GeoMean, geometric mean; IFNγ, interferon gamma; mBMC, mixed bone marrow chimera; MDAM, ASNENMDAM; METM, ASNENMETM; PR8, influenza A strain PR/8/34; WT, wild-type. (TIF) [file pbio.3000648.s008.tif]

Gel 1 – 700nm exposure

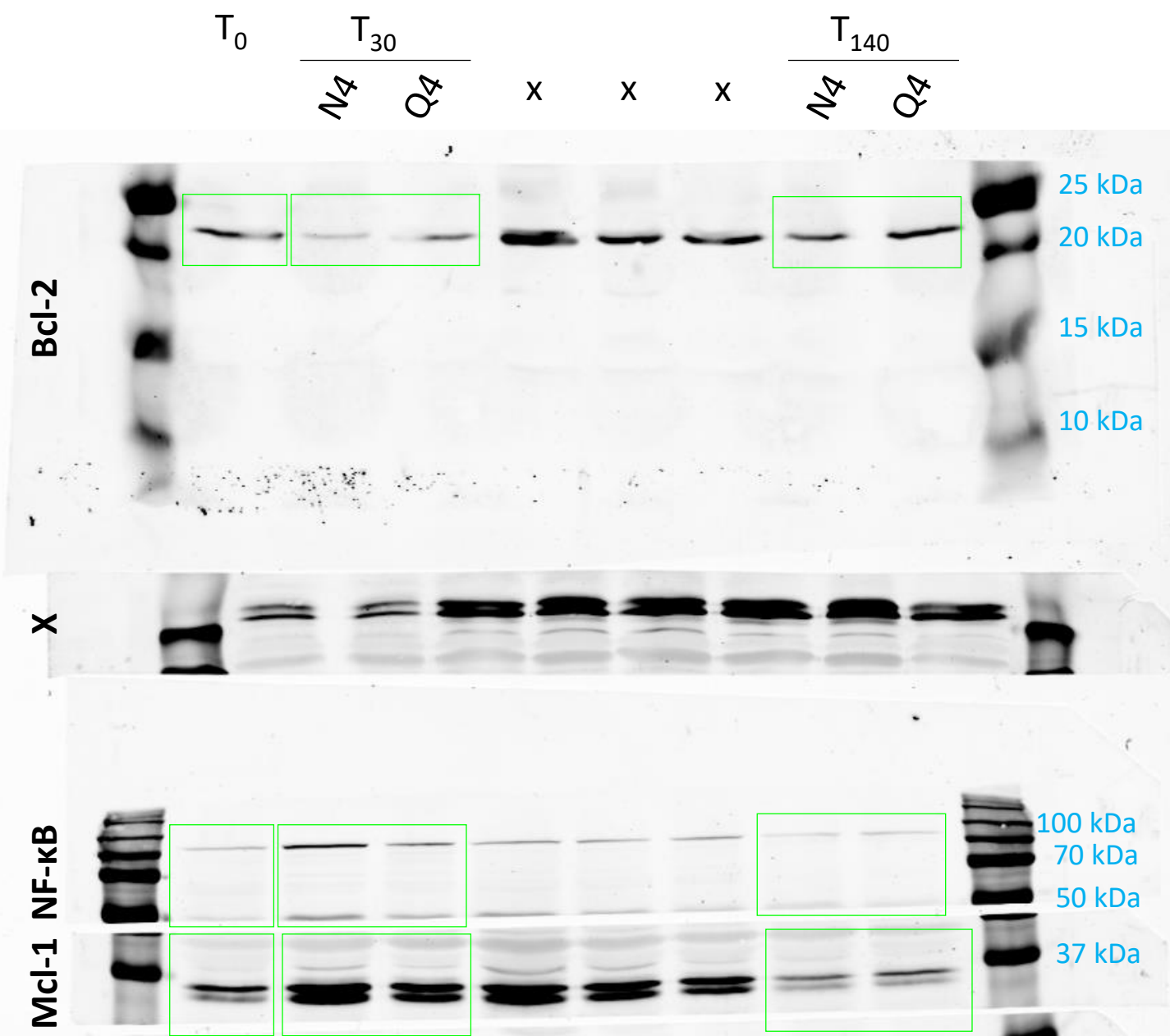

Gel 1 – 800nm exposure

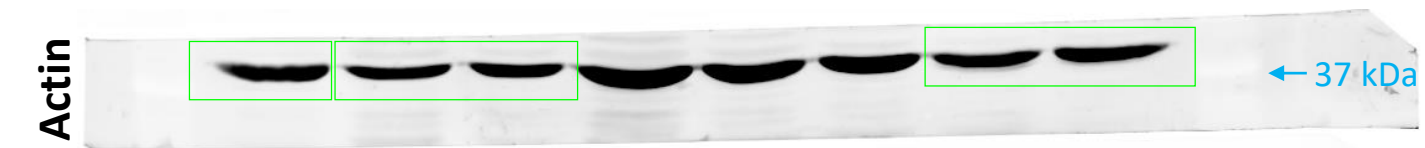

Gel 2 – 700nm exposure

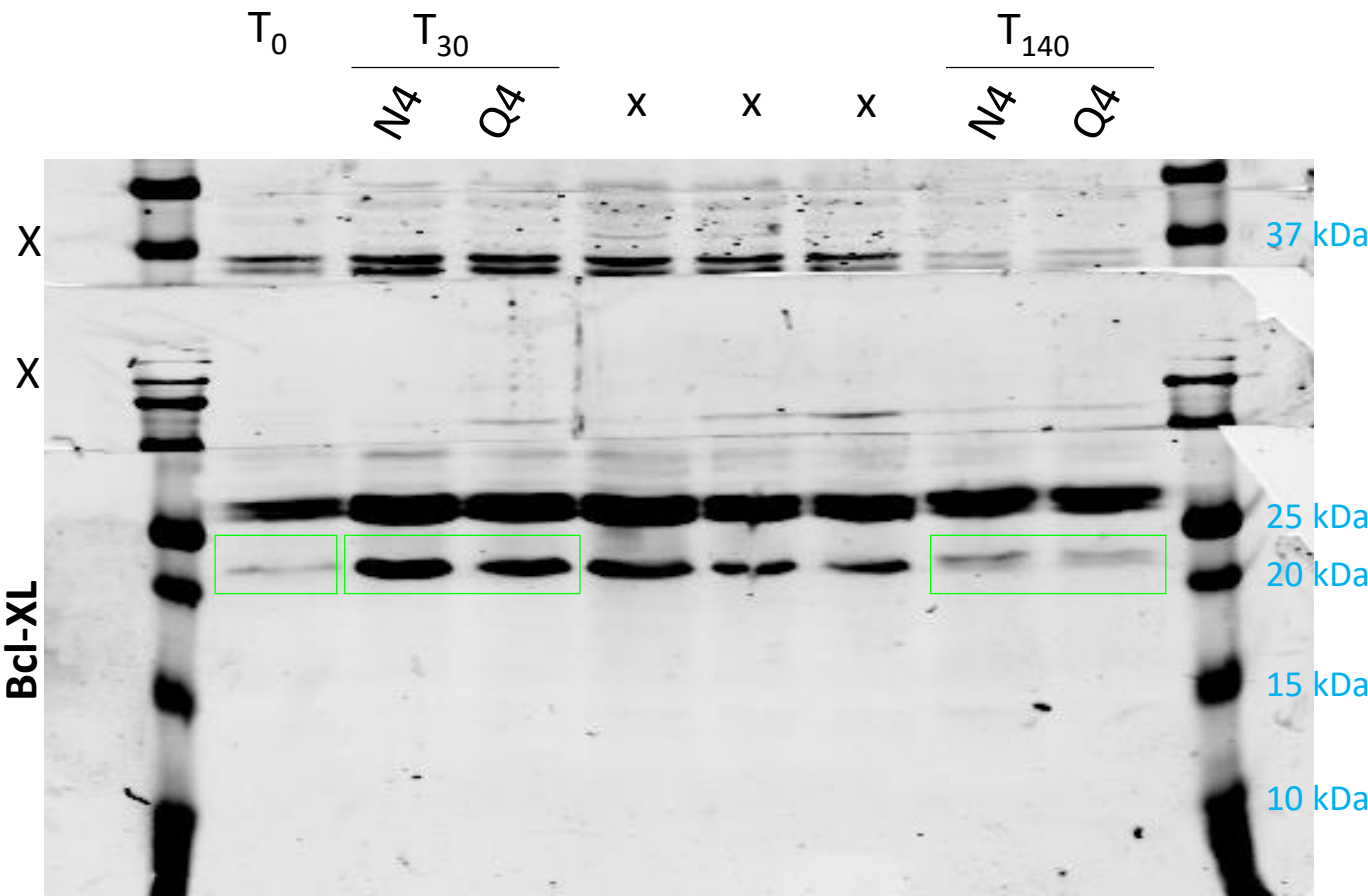

Gel 2 – 800nm exposure

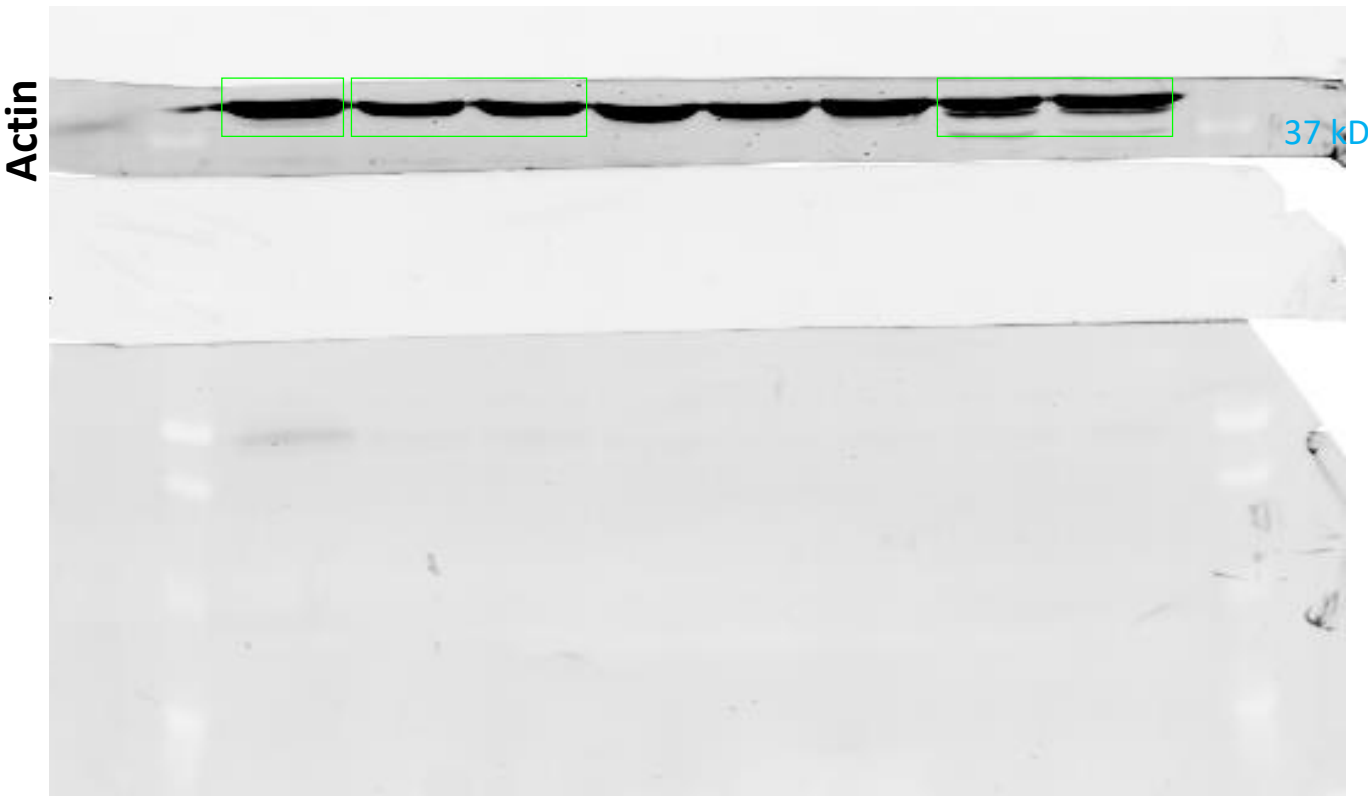

Gel 3 – 700nm exposure

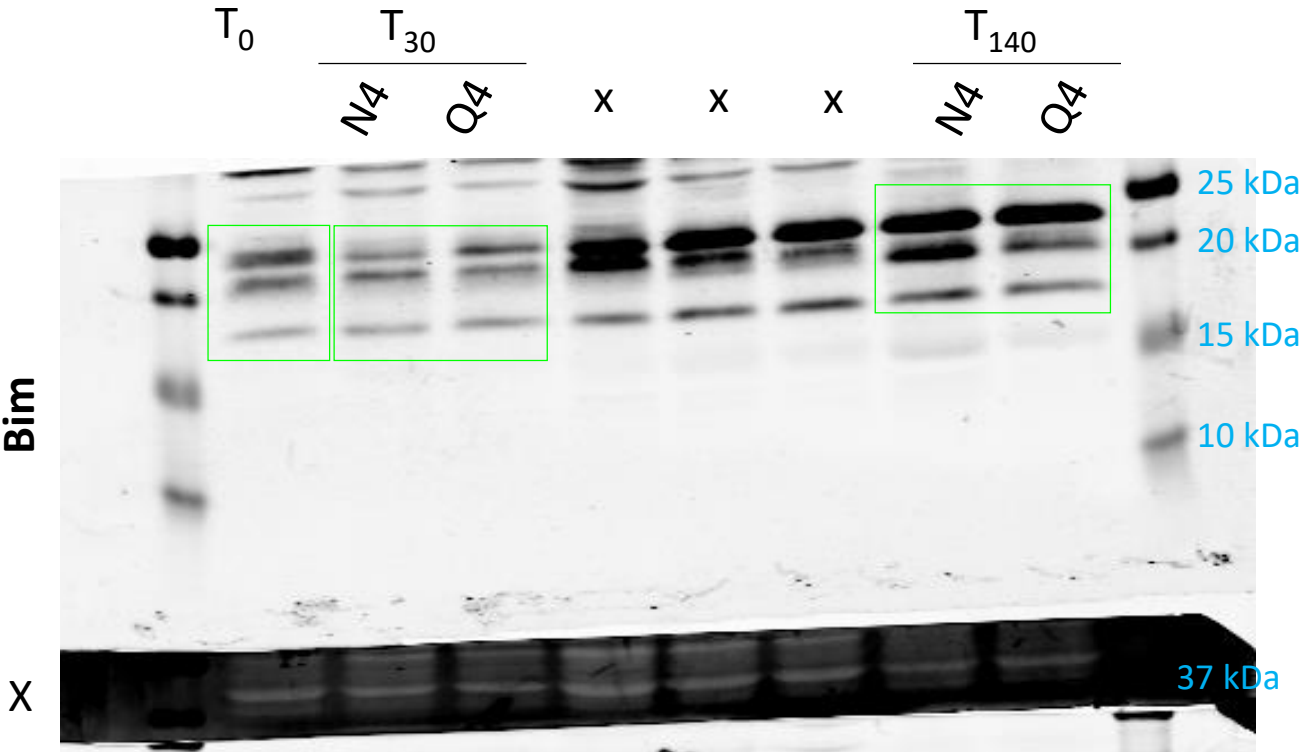

Gel 3 – 800nm exposure

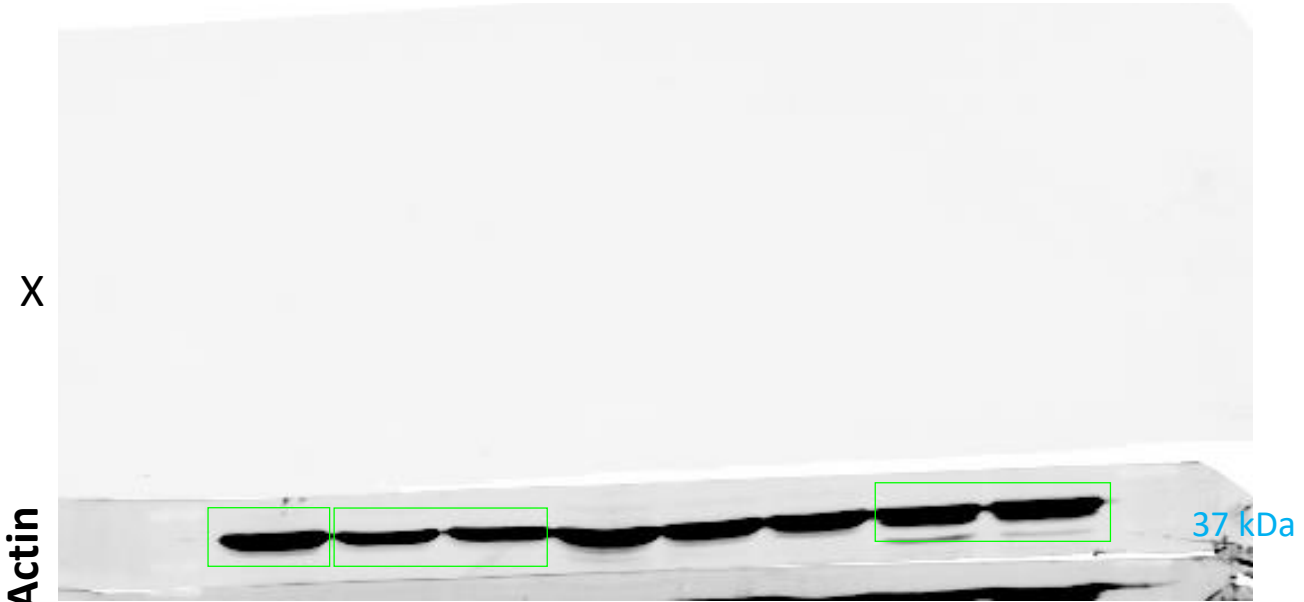

Supplement: S1 Raw Images — Samples for WB were lysed in Laemmli lysis buffer (0.12 M Tris HCL [pH 8], 4% SDS, 20% glycerol, 0.05 μg/μL bromophenol blue, and 50 mM dithiothreitol) and boiled for 5 min. Protein contents were determined by the Bio-Rad protein assay (Bio-Rad Laboratories), and equal amounts of total lysate were analyzed by 12% SDS–polyacrylamide gel electrophoresis. Each sample was loaded on three separate blots. Proteins were transferred to Immobilon-P and incubated with blocking buffer (TBS-T) containing 2% low-fat milk for 1 hour. Blots were then cut according to protein markers to allow analysis of multiple proteins from a single membrane. Next, membranes were incubated with an antibody against Mcl-1 (BD Pharmingen), Bcl-XL (Transduction Laboratories), Bim (Stressgen Bioreagents), Bcl-2 (Enzo Lifesciences), p100/p52 (Cell Signaling), or β-actin (Santa Cruz Biotechnology) overnight at 4 °C in TBS-T. Blots labeled with β-actin were incubated with IRDye 800. All other antibodies were labeled with IRDye 680 (Both Li-Cor) for 1 hour. Odyssey Imager (Li-Cor) was used as a detection method. IRDye 680 was visualized at 700 nm and IRDye 800 at 800 nm. “X” indicates irrelevant sample or irrelevant membrane. TBS-T, Tris buffered saline/Tween-20; WB, western blot. (PDF) [file pbio.3000648.s010.pdf]
